# Valorization of Seafood Processing Wastes Using Subcritical Water Extraction—A Comprehensive Review

**DOI:** 10.3390/md24090307

**Published:** 2026-09-02

**Authors:** Laleh Nazari, Melissa Kosik

**Affiliations:** Aquatic and Crop Resource Development Research Centre, National Research Council of Canada, 1411 Oxford Street, Halifax, NS B3H 3Z1, Canada; melissa.kosik@nrc-cnrc.gc.ca

**Keywords:** seafood processing waste, subcritical water extraction, hydrothermal processing, waste valorization, seafood biorefinery, bioactive peptides, protein hydrolysates, omega-3 fatty acids, circular bioeconomy

## Abstract

**Highlights:**

**What are the main findings?**

- Seafood processing by-products are rich resources of proteins, lipids, collagen, chitin, and minerals.
- Subcritical water extraction provides a green platform for seafood by-product valorization.
- Process conditions strongly influence extraction efficiency and product quality.
- Subcritical water extraction enables recovery of proteins, peptides, amino acids, oils and minerals.

**What are the implications of the main findings?**

- Integrated green technologies can improve seafood biorefinery performance.
- Scale-up, reactor cost, product stability, and downstream processing remain key challenges.

**Abstract:**

The global seafood industry generates substantial quantities of processing by-products such as heads, viscera, skin, bones, scales, and shells. These residues represent an underutilized resource rich in proteins, lipids, minerals, enzymes, and polysaccharides. Conventional valorization approaches such as chemical extraction, wet rendering, and enzymatic hydrolysis have been used to recover valuable compounds from seafood waste. However, conventional methods often involve high chemical consumption, long processing times, and environmental concerns. Green extraction technologies have emerged as promising alternatives, with subcritical water extraction (SWE) gaining significant attention due to its unique properties and ability to simultaneously extract and convert biomass components. This review provides a comprehensive overview of the valorization of seafood processing wastes using SWE. Particular emphasis is placed on the physicochemical properties of subcritical water, the reaction mechanisms governing the hydrolysis and transformation of proteins, lipids, and polysaccharides, and the key parameters influencing extraction performance. Recent advances in the recovery of value-added products such as amino acids, bioactive peptides, protein hydrolysates, omega-3-rich oils, chitin derivatives, and mineral-rich materials are summarized. In addition, the integration of SWE with complementary technologies such as supercritical CO_2_ extraction, enzymatic hydrolysis, and hydrothermal carbonization is examined as a strategy for developing integrated seafood biorefineries.

## 1. Introduction

The seafood industry, a vital component of global food production, sustains millions of livelihoods and provides a significant source of essential nutrients, such as protein, vitamins and omega-3 fatty acids for human consumption. According to the Food and Agricultural Organization (FAO) of the United Nations, global seafood production from fisheries and aquaculture has seen a substantial increase from 134.4 million tonnes in the 2000s to 194.6 million tonnes in 2024. This growth can be attributed to the advancements in fishing technologies and the rapid progress in aquaculture [1,2]. The expansion in seafood production has led to a surge in by-products and waste materials, constituting a substantial portion, typically ranging from 30% to 70%, of the total seafood output following industrial processing [3]. The by-products have a heterogenous nature and include solid discards such as bones, head, skin, scales, fins, tails, and viscera as well as wastewaters. Some low-economic value seafood and undersized fish or by-catch that are unsuitable for human consumption are also discarded [3]. These by-products are an important source of valuable compounds such as proteins, fatty acids and minerals, as their composition is similar to that of fish products used for consumption. Thus, discarding them represents a huge economic loss due to the loss of nutrients as well as environmental concerns associated with water and land contamination [1]. In addition to the solid waste, the aquaculture and seafood industry generates significant volumes of wastewater containing organic components like uneaten food and faeces, along with nutrients such as phosphorous and nitrogen [4,5]. Historically, seafood waste has been partially repurposed for fish meal, fertilizers, animal feed and fish oil with limited profitability, or it has been discarded into the environment, contributing to pollution problems and resource wastage [1,3]. To address these issues, the valorization of seafood discards through the extraction of valuable compounds or their conversion into profitable products holds the potential for significant environmental and economic benefits, as well as improved waste management.

Conventional methods for valorizing seafood waste have relied on physical and chemical processes, including acid/alkali and solvent extraction [4]. However, these methods have notable limitations such as the use of hazardous chemicals and solvents, low extraction efficiency, time-consuming procedures, environmental pollution, and potential health hazards for operators. Consequently, there has been a growing interest in alternative methods, particularly green extraction technologies [3]. These technologies include enzymatic-assisted extraction, fermentative extraction, microwave-assisted extraction, ultrasound-assisted extraction, supercritical-fluid-assisted extraction and subcritical water extraction [3].

Of these alternatives, subcritical water has gained considerable attention as a green solvent for valorizing various waste streams, including seafood processing by-products. This emerging technology leverages the unique properties of water under controlled temperature and pressure conditions, offering a versatile platform for converting seafood waste materials into valuable products. Subcritical water, operating between 100 °C and 374 °C under pressures sufficient to maintain water in the liquid state (typically 10–220 bar depending on temperature), demonstrates remarkable solvent capabilities, enabling the efficient extraction of bioactive compounds, the conversion of organic matter into biofuels, and the production of high-value chemicals from seafood waste materials [3,6]. The utilization of subcritical water not only mitigates the environmental impact of seafood processing waste but also creates new economic opportunities, reducing the overall cost burden on the industry.

In recent years, extensive research has been conducted to explore the potential of SWE for valorizing seafood processing waste, leading to significant advancements in this field [6,7,8,9,10,11,12,13]. Yet, a comprehensive review that synthesizes the key findings, methodologies, and challenges in this field is currently lacking. This review paper aims to bridge this gap by providing a detailed overview of the various subcritical water-based approaches employed for the valorization of seafood processing wastes. Through the critical analysis of the existing literature, this review elucidates the scientific and technological advancements, highlights potential barriers, and offers insights into the future directions of this exciting and environmentally conscious endeavor.

To ensure transparency and reproducibility, the literature included in this review was identified through comprehensive searches of major scientific databases, including Scopus, Web of Science, ScienceDirect, and Google Scholar. Searches were conducted using combinations of keywords such as seafood waste, seafood processing by-products, fish waste, shellfish waste, subcritical water extraction, subcritical water, hydrothermal processing, hydrothermal liquefaction, hydrothermal carbonization, protein hydrolysates, lipid extraction, bioactive compounds, biorefinery, and valorization. Publications published primarily between 2000 and 2026 were considered, while earlier landmark studies were included where necessary to provide historical context. Peer-reviewed journal articles were prioritized, together with selected review articles and authoritative reports. Studies were included if they addressed seafood processing wastes, subcritical water processing, reaction mechanisms, process optimization, product recovery, or related hydrothermal technologies relevant to the objectives of this review. Publications lacking sufficient technical information or relevance to the scope of the review were excluded.

This review investigates the principles of SWE, explores the diverse seafood waste streams that can be valorized using this approach, highlights the valuable products that can be obtained, and discusses the sustainability implications of adopting these methods. To illustrate the scale and characteristics of seafood processing residues and the opportunities for their valorization, the Canadian seafood sector is presented as an illustrative case study. The review further examines the challenges and limitations associated with SWE and identifies key research gaps and opportunities for future development. By providing a comprehensive synthesis of current knowledge, this review aims to support further research, innovation, and the implementation of sustainable valorization strategies within the seafood industry and related sectors.

## 2. Canadian Fisheries and Aquaculture: An Illustrative Case Study

Although this review focuses on global advances in seafood waste valorization using subcritical water technologies, Canada is presented as an illustrative case study due to its large fisheries and aquaculture sectors and the substantial quantities of seafood processing by-products generated annually.

The fishing industry is one of the oldest industries in Canada. The primary fisheries of Canada are located along the Atlantic and Pacific coasts, as well as smaller fisheries in the Arctic region. Atlantic provinces with major fisheries include Newfoundland and Labrador, Prince Edward Island, New Brunswick, and Nova Scotia. The major Pacific fisheries are in British Columbia [14]. In 2024, the total landings from sea fisheries and freshwater fisheries were 626,225 and 24,888 tonnes, respectively. Table 1 presents a regional overview of fisheries landings and aquaculture production in Canada in 2024, highlighting the large scale of the Canadian seafood industry [15]. Atlantic Canada accounted for the largest proportion of sea fisheries landings, with 546,098 tonnes valued at approximately CAD 3.63 billion, demonstrating the major contribution of this region to the national seafood sector. Freshwater fisheries contributed 23,160 tonnes nationally, with Ontario recording the highest catches and landings at 11,821 tonnes, mainly consisting of yellow pickerel, perch, smelt, and whitefish [16,17]. Table 2 shows the commercial sea fisheries landings by species group in 2024, with shellfish representing the dominant category in Atlantic Canada.

The high production volumes associated with seafood processing activities generate substantial quantities of by-products and waste streams, including fish heads, viscera, skin, bones, shells, scales, and processing wastewater. These residues are rich in proteins, lipids, minerals, and polysaccharides, and therefore represent valuable biomass resources for recovery of bioactive compounds and bioenergy production. The large quantities of seafood processing residues generated annually in Canada, particularly in Atlantic provinces, create significant potential for the application of valorization technologies.

**Figure 1:**
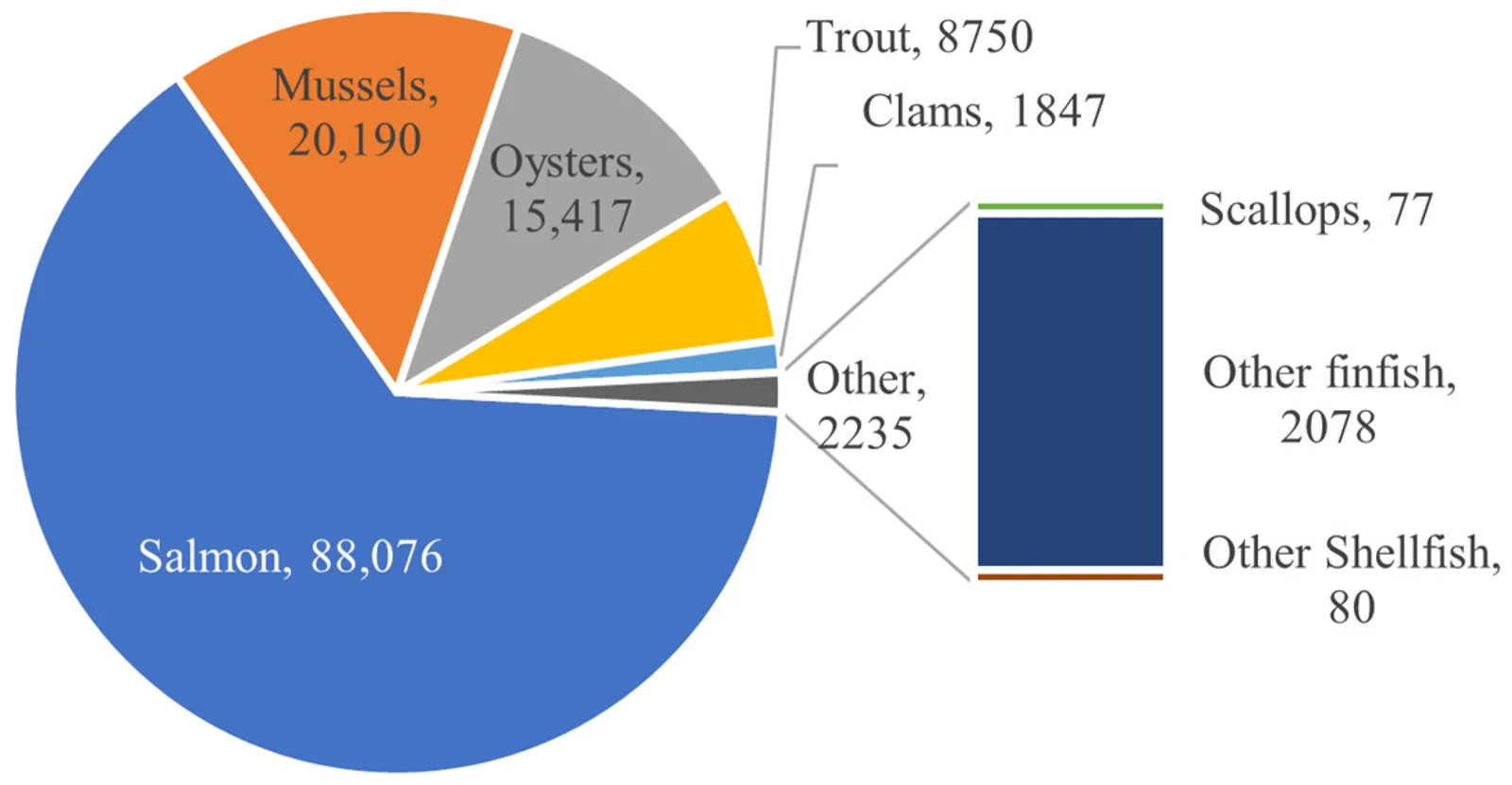
Canadian aquaculture production in 2024 by species, based on total harvest weight (tonnes).

The aquaculture sector in Canada has been active since the 1980s and has expanded considerably over recent decades. Aquaculture is practised in all provinces and in Yukon; however, the major operations are concentrated in British Columbia (40.0% of total production), followed by New Brunswick (17.4%), Newfoundland and Labrador (16.3%), and Prince Edward Island (13.1%), based on 2024 data [14,18]. As shown in Table 1, Canada had 615 aquaculture establishments in 2024, producing a total of 160,318 tonnes of seafood valued at approximately CAD 1.36 billion. Atlantic salmon is the most produced and economically valuable aquaculture species, primarily cultivated in the coastal waters of British Columbia, New Brunswick, and Nova Scotia, followed by mussels, oysters and trout [18]. The continuous growth of aquaculture production has also increased the generation of organic side streams and processing residues that require sustainable management and valorization strategies. Figure 1 shows the aquaculture production volume by species in 2024 [18].

### Atlantic Fisheries and Aquaculture

Atlantic Canada plays a dominant role in the Canadian seafood sector due to its extensive coastline, abundant marine resources, and well-established fisheries and aquaculture industries. The Atlantic provinces, including Newfoundland and Labrador, Prince Edward Island, Nova Scotia, and New Brunswick, account for the majority of Canada’s commercial seafood landings and a significant portion of national aquaculture production [15,16]. The cold, nutrient-rich waters of the Atlantic Ocean support commercially important species such as lobster, snow crab, shrimp, scallops, herring, Atlantic salmon, mussels, and oysters.

In 2024, Atlantic Canada accounted for approximately 546,098 tonnes of commercial fisheries landings, representing the largest regional contribution to Canada’s seafood industry [15]. Shellfish dominated the landings, particularly lobster and snow crab, which are among the highest-value seafood products in Canada. In addition, Atlantic Canada contributes substantially to the national aquaculture sector, with major production concentrated in Atlantic salmon, mussels, and oysters [16,18]. More than 50% of Canada’s aquaculture production originates from Atlantic Canada, where over 70 aquatic species are licensed for farming [19].

The large-scale harvesting, farming, and processing of seafood in Atlantic Canada generates significant quantities of by-products and waste streams, including heads, frames, viscera, shells, skin, scales, and processing wastewater. These residues are rich in proteins, lipids, minerals, collagen, chitin, and other bioactive compounds, creating strong opportunities for valorization through sustainable technologies such as subcritical water extraction and hydrothermal processing. Therefore, the Atlantic seafood sector represents an important feedstock source for the development of integrated seafood waste biorefineries and circular bioeconomy approaches.

## 3. Seafood By-Products and Their Composition

Landed seafood needs to undergo processing to make the product market-friendly and consumable, while also maintaining the physicochemical quality of the product. Seafood primary processing operations include washing, heading and gutting, scaling/shell removal, and filleting, skinning, trimming and deboning, which generate substantial quantities of solid and liquid by-products [14,20]. Solid by-products include fish parts like the head, frame, and viscera, as well as crustacean shells (e.g., crab carapace and shrimp shells), and mollusk by-products (e.g., mussel, oyster and clam shells). Liquid by-products typically refer to the blood and other fluids, and the wastewater generated during processing [14]. Table 3 presents the approximate proportions of seafood by-products and their major valuable components [21,22,23].

Seafood by-products contain a wide range of recoverable bioactive compounds and biomolecules with significant industrial and commercial value [24]. These by-products are rich in proteins, lipids, minerals, enzymes, and polysaccharides that can be converted into high-value products for food, nutraceutical, pharmaceutical, agricultural, and biomedical applications. Table 4 summarizes the typical composition of the by-products from fish, crustaceans, mollusks, and other marine organisms [4,20].

The high moisture content and abundance of proteins, lipids, minerals, and biopolymers make seafood by-products particularly suitable feedstocks for subcritical water and hydrothermal processing technologies. Under subcritical water conditions, these biomolecules can undergo hydrolysis, solubilization, and conversion into value-added products such as peptides, amino acids, fish oils, organic acids, hydroxyapatite, and bioactive compounds. The major recoverable components present in seafood waste, including proteins, lipids, enzymes, minerals, and polysaccharides, are discussed in the following sections.

### 3.1. Proteins

Seafood by-products are a considerable source of valuable proteins, typically accounting for 15–30% of the total by-product weight, and have a well-balanced essential amino acid composition. Major protein-derived products obtained from seafood by-products include fish protein hydrolysates, bioactive peptides, collagen, and gelatin [21,25]. Due to their susceptibility to hydrolysis under hydrothermal conditions, proteins are among the most extensively studied compounds in subcritical water processing.

Fish protein hydrolysates are a mixture of peptides and amino acids produced through partial or complete hydrolysis of proteins. They are characterized by high digestibility, excellent solubility, and various bioactive properties such as antioxidant and antihypertensive activities [25]. Similarly, bioactive peptides released from fish proteins exhibit antioxidant, antihypertensive, antimicrobial, and anti-inflammatory effects, making them attractive for food, nutraceutical, and pharmaceutical applications [26]. Conventional production methods include enzymatic hydrolysis, acid/alkaline extraction, fermentation, ultrasound-assisted extraction, microwave-assisted extraction, and solvent-based techniques [25]. However, many of these methods rely on substantial chemical usage, long processing times, or expensive enzymes. SWE has emerged as a promising alternative because the elevated ionic product of water under subcritical conditions promotes protein hydrolysis without the need for additional catalysts or other inputs. Nevertheless, severe hydrothermal conditions (>250 °C) may lead to amino acid degradation and Maillard-type reactions, highlighting the importance of optimizing SWE operating conditions.

Collagen is the major structural protein found in fish skin, bones, and connective tissues, representing approximately 20–30% of the total fish protein content [25,27]. Gelatin is produced by the partial hydrolysis and denaturation of collagen, and is widely used in the food, cosmetic, pharmaceutical, and biomedical industries [20]. Compared to mammalian collagen, fish-derived collagen generally contains lower fat levels, exhibits higher digestibility, and presents a reduced risk of transmitting infectious diseases, making it a safer and more sustainable alternative [27,28]. Fish gelatin also possesses desirable properties such as biodegradability, film-forming ability, and antioxidant activity, making it suitable for medical, food, and pharmaceutical applications [28]. Extraction methods include acid treatment, enzymatic hydrolysis, thermal processing, ultrasound-assisted extraction, and high-pressure processing. Recently, subcritical water processing has attracted considerable interest as a greener alternative capable of simultaneously hydrolyzing collagen and producing bioactive peptides.

### 3.2. Lipids

The lipid content of fish by-products ranges from 2% to 30% depending on the fish species, age, sex, season and the distribution of fat within different body parts [25]. Fish lipids are primarily concentrated in viscera and heads, although smaller amounts are also present in other parts such as skin, fins, frames, and tails. Fish oil contains triglycerides, phospholipids, free fatty acids, sterol esters, and fat-soluble vitamins such as vitamins A and D. The fatty acids in fish oil include saturated (SFA), monounsaturated (MUFA), and polyunsaturated fatty acids (PUFA) [29,30].

Among these compounds, the omega-3 polyunsaturated fatty acids eicosapentaenoic acid (EPA) and docosahexaenoic acid (DHA), are the most nutritionally valuable components due to their cardiovascular, anti-inflammatory, and neurodevelopmental health benefits [20,25,27]. Since the human body is unable to synthesize sufficient quantities of omega-3 fatty acids, they must be obtained through dietary sources.

Fish oil extraction techniques include solvent extraction, mechanical pressing, enzymatic hydrolysis, microwave-assisted extraction, ultrasound-assisted extraction, and supercritical fluid extraction [25]. However, many conventional methods require large volumes of organic solvents, extended extraction times, or intensive pre-treatment steps. Subcritical water processing has gained attention as a greener alternative capable of simultaneously disrupting tissues and enhancing lipid release.

### 3.3. Enzymes

Fish processing by-products, particularly viscera and other internal organs, are rich sources of industrially important enzymes such as proteases, transglutaminases (TGases), lipases, and carbohydrases [20,25,27]. Common enzymes isolated from fish waste include pepsin, trypsin and chymotrypsin, which are widely used in food processing, collagen extraction, protein hydrolysis, and biotechnological applications [25].

Fish species adapted to cold marine environments often produce enzymes with high catalytic activity at relatively low temperatures (20–25 °C), making them particularly attractive for food processing applications where mild operating conditions are preferred [22,23]. Conventional enzyme recovery methods include salt precipitation, isoelectric solubilization/precipitation, ultrafiltration, membrane filtration, flocculation, and solvent extraction [31].

Although seafood by-products represent valuable enzyme sources, the elevated temperatures typically employed during SWE often lead to enzyme denaturation and loss of catalytic activity. Consequently, SWE is generally more suitable for the hydrolysis and conversion of proteins rather than direct enzyme recovery.

### 3.4. Minerals

Fish processing waste, particularly bones, heads and scales, contains a variety of essential minerals that support nutrient recycling and sustainable agricultural practices. These mineral-rich streams can help reduce our dependence on non-renewable nutrient resources.

Fish bone typically consists of approximately 30% collagen and 60–70% minerals, primarily calcium and phosphorous [20,25]. The phosphorous is mainly present in the form of calcium phosphate, which is an essential nutrient for agricultural fertilization [32]. In addition, hydroxyapatite recovered from fish bones has significant biomedical applications because of its excellent biocompatibility, mechanical stability, and bone-bonding properties under physiological conditions [20,25]. Due to the increasing demand for sustainable calcium sources, fish-derived bio-calcium has attracted considerable attention as an alternative to conventional calcium supplements such as calcium carbonate and calcium citrate [20,33]. Fish bones and scales may also contain appreciable amounts of iodine, magnesium, and selenium [25].

Compared with conventional acid-based mineral extraction processes, SWE offers an environmentally friendly alternative for recovering mineral-rich fractions while simultaneously hydrolyzing organic matter. Furthermore, integrating mineral recovery with protein and lipid extraction supports the development of zero-waste seafood biorefineries.

### 3.5. Polysaccharides

Seafood processing waste contains various polysaccharides, mainly derived from skin, scales, and connective tissues. Among the most important marine polysaccharides are chitin and glycosaminoglycans (GAGs), the latter comprising both non-sulfated and sulfated polysaccharides.

Chitin is a structural polysaccharide abundantly present in the shells of crustaceans, such as shrimp, crab, and lobster. Conventional chitin extraction typically involves demineralization using dilute hydrochloric acid followed by deproteinization with sodium hydroxide. However, these chemical-intensive processes generate large quantities of wastewater and may negatively affect product quality. Consequently, environmentally friendly alternatives such as hydrothermal and subcritical water-assisted processing have attracted increasing interest.

Chitosan is produced through the deacetylation of chitin using alkaline treatment. Due to its antimicrobial activity, biodegradability, and film-forming properties, chitosan has numerous applications in food packaging, biomedical materials, pharmaceuticals, and wastewater treatment [25,27]. Chitin and chitosan can also serve as precursors for producing various bioactive compounds, including antioxidants, antidiabetic agents, and functional biomaterials [23,27].

GAGs are long, unbranched polysaccharides composed of repeating disaccharide units and are found primarily in fish connective tissues. Most GAGs carry a negative charge due to the presence of sulfate and/or carboxyl groups and are widely used in joint health supplements [34,35]. GAGs comprise both non-sulfated and sulfated polysaccharides. Hyaluronic acid (HA) is the principal non-sulfated GAG, whereas heparin, heparan sulfate, dermatan sulfate, chondroitin sulfate, and keratan sulfate are sulfated GAGs. Although present at relatively low concentrations, these carbohydrates have considerable economic value due to their bioactivity and high market demand. GAGs possess important biological functions and are widely used as anticoagulants, lubricating agents, and wound-healing materials. Traditionally, these compounds have been isolated from terrestrial slaughterhouse waste (predominantly porcine, bovine, and poultry); however, increasing concerns regarding bovine spongiform encephalopathy and sustainability have stimulated interest in marine-derived alternatives [23].

Overall, marine polysaccharides represent valuable biopolymers with significant industrial potential. The development of greener extraction technologies, including subcritical water processing, may reduce chemical consumption while improving the sustainability of marine biopolymer recovery.

## 4. Conventional Methods for Valorization of Seafood Waste

Conventional valorization techniques for seafood processing waste mainly involve physical, chemical, biological, and biochemical processes for recovering lipids, proteins, and minerals. Chemical methods are the most commonly used techniques employed to recover lipids, proteins, collagen, chitin, minerals, and other bioactive compounds. These conventional methods are widely established at both bench and industrial scales and have been extensively used to convert seafood by-products into value-added products. However, many conventional extraction techniques are associated with drawbacks such as high chemical and energy consumption, long processing times, generation of hazardous waste, and degradation of heat-sensitive compounds. These limitations have driven increasing interest in greener and more sustainable technologies, including SWE, for seafood waste valorization.

### 4.1. Chemical Methods

Chemical extraction methods involve the use of acids, alkalis, and organic solvents to break down and rupture the cellular structure and recover lipids, proteins, collagen, chitin, and minerals from seafood waste. These methods generally provide high extraction efficiencies; however, they often require large quantities of hazardous chemicals and subsequent purification steps, raising environmental and economic concerns.

#### 4.1.1. Acid/Alkaline Extraction

Acid and alkaline hydrolysis are widely used for the recovery of proteins, amino acids, collagen, gelatin, chitin, chitosan, and minerals from seafood by-products. Strong acids or alkalis hydrolyze proteins into smaller peptides and amino acids and assist in the removal of minerals, pigments, and other non-target components. Acid treatment is also commonly used as a pre-treatment step prior to enzymatic hydrolysis to improve extraction yield [36].

Acidic and alkaline treatments are extensively applied in chitin extraction from crustacean shells through demineralization and deproteinization processes. Similarly, collagen and gelatin can be recovered from fish skin, scales, and bones using dilute organic acids, which enhance collagen solubility by disrupting intermolecular interactions, facilitating the release of collagen from the surrounding matrix while largely preserving its native triple-helical structure under controlled extraction conditions [23,37].

Although these methods are effective and widely used, they may alter or degrade sensitive amino acids and bioactive compounds, generate large amounts of chemical waste, and require neutralization and purification steps after extraction. These limitations have encouraged the development of greener alternatives such as SWE, which can hydrolyze and recover valuable compounds with reduced chemical usage.

#### 4.1.2. Organic Solvent Extraction

Organic solvents are primarily used for lipid extraction from seafood by-products. Common solvents include hexane, chloroform–methanol mixtures, acetone, cyclohexane, and petroleum ether. These solvents dissolve lipids by disrupting interactions between lipids and the cellular matrix [36].

Among the most widely used methods are the Folch or Bligh and Dyer methods, both based on partitionable mixtures of polar and non-polar solvents, for efficient extraction of total lipids. Soxhlet extraction is another commonly used technique, particularly for analytical applications, although it generally requires larger solvent volumes and longer extraction times [36,38].

Solvent extraction methods are valued for their high lipid recovery efficiency; however, the use of toxic and flammable solvents raises environmental, health, and safety concerns. In addition, solvent recovery and purification increase processing costs and energy demand. These disadvantages have promoted interest in alternative extraction technologies such as SWE, where water can act as a tunable solvent under elevated temperature and pressure conditions.

### 4.2. Physical Methods

Physical and mechanical methods are mainly based on mechanical separation, heating, or size reduction processes, and are commonly used for fish oil and fishmeal production. These techniques are generally simple, cost-effective, and suitable for industrial-scale operations; however, they may lead to the degradation of thermally sensitive compounds and often provide lower selectivity than chemical or biological methods.

#### 4.2.1. Wet Rendering

Wet rendering is one of the most widely used industrial methods for fish oil extraction. In this process, seafood by-products are cooked at 90–100 °C at atmospheric pressure to coagulate proteins, followed by mechanical pressing to separate the oil and aqueous phases from the solids. The remaining solids are typically dried and processed into fishmeal [36,39].

Although wet rendering is economically attractive and suitable for large-scale production, the combination of thermal processing and the presence of oxygen may accelerate lipid oxidation and degradation of oxidation-sensitive compounds such as the omega-3 fatty acids EPA and DHA.

#### 4.2.2. Maceration

Maceration involves soaking seafood by-products in a solvent under mild conditions to facilitate the diffusion of soluble compounds into the liquid phase. This technique is commonly used for recovering pigments, lipids, and other bioactive compounds.

The method is relatively simple and can be performed at low temperatures, reducing thermal degradation of sensitive compounds. However, extraction efficiency is often limited by long extraction times and high solvent consumption [37].

#### 4.2.3. Cold Pressing

Cold pressing, also known as mechanical expression, extracts oils and liquids by mechanically pressing seafood tissues without applying significant heat. Compared with wet rendering, cold pressing better preserves the nutritional and functional quality of sensitive fatty acids [37]. Despite these advantages, the method generally produces lower extraction yields and may leave substantial residual oil in the solid fraction. Consequently, cold pressing is often combined with other extraction methods to improve recovery efficiency.

### 4.3. Biological/Biochemical Methods

Biological and biochemical methods focus on using biological agents such as enzymes or microorganisms to break down the tissues and release valuable components such as proteins, peptides, lipids and collagen. Compared with chemical extraction, these approaches operate under milder conditions and can improve the functional and nutritional quality of the recovered products.

#### 4.3.1. Enzymatic Hydrolysis

Enzymatic hydrolysis is one of the most widely studied biological methods for seafood waste valorization. Proteolytic enzymes such as Alcalase, pepsin, and trypsin hydrolyze proteins into smaller peptides or amino acids, generating fish protein hydrolysates. This process can also facilitate lipid release from fish tissues [39,40].

The efficiency of enzymatic hydrolysis depends on several factors, including enzyme type, enzyme concentration, enzyme-to-substrate ratio, pH, temperature, and reaction time. This method offers important advantages such as high selectivity, mild operating conditions, and reduced use of harsh chemicals. However, enzymatic hydrolysis is often associated with relatively high enzyme costs, long processing times, and challenges in industrial-scale implementation [41].

#### 4.3.2. Fermentation

Fermentation utilizes microorganisms to convert seafood by-products into value-added products, including protein hydrolysates, enzymes, biofuels, chitin, and chitosan. During fermentation, microorganisms produce extracellular enzymes that degrade complex organic materials into simpler compounds [36,40,41].

Fermentation is considered an environmentally friendly process due to its mild operating conditions and reduced chemical requirements. Nevertheless, the process can be time-consuming and requires careful optimization of microbial strains and operating conditions. In addition, maintaining consistent product quality at industrial scale remains challenging [41].

Table 5 presents selected examples of conventional extraction methods used for seafood waste valorization.

## 5. Advanced and Emerging Green Technologies for Seafood Waste Valorization

The growing environmental concerns associated with conventional seafood waste processing methods have accelerated the development of advanced green extraction technologies. These approaches aim to improve extraction efficiency while reducing solvent consumption, energy demand, processing time, and environmental impact. Emerging technologies, including supercritical fluid extraction (SFE), pulsed electric field (PEF), ultrasound-assisted extraction (UAE), microwave-assisted extraction (MAE), and cold plasma, have shown considerable potential for recovering high-value compounds such as lipids, proteins, peptides, pigments, collagen, and other bioactive molecules from seafood by-products. Compared with conventional extraction methods, these technologies generally operate under milder or more controlled conditions and can improve product quality and process sustainability. Table 6 summarizes representative applications of emerging technologies for the recovery of value-added compounds from seafood processing by-products.

### 5.1. Supercritical Fluid Extraction (SFE)

Supercritical fluid extraction utilizes fluids at temperatures and pressures above their respective critical points to recover valuable compounds from biomass. Under supercritical conditions, solvents exhibit gas-like diffusivity, along with the viscosity and flow behaviour of liquids, thereby facilitating the extraction of target compounds. Carbon dioxide (CO_2_) is the most commonly used solvent due to its non-toxicity, low critical temperature, and ease of removal from the final product. SFE has been extensively investigated for the extraction of high-quality fish oils rich in omega-3 fatty acids from seafood by-products. In addition to improving lipid quality and oxidative stability, SFE can reduce the use of hazardous organic solvents and minimize subsequent refining requirements. However, the process often requires expensive high-pressure equipment and relatively high operational costs [36,55].

### 5.2. Pulsed Electric Field Extraction (PEF)

Pulsed electric field extraction is a non-thermal technology that applies short high-voltage pulses to biological materials, resulting in electroporation of cell membranes and enhanced release of intracellular compounds. This method has been explored for improving the extraction of lipids, pigments, and other bioactive compounds from seafood side streams, while reducing thermal degradation and processing time. Nevertheless, industrial-scale implementation of PEF remains limited, and process efficiency is influenced by several operational and material-related factors [36,56].

### 5.3. Ultrasound-Assisted Extraction (UAE)

Ultrasound-assisted extraction employs acoustic cavitation to disrupt cell structures and enhance mass transfer, thereby improving the recovery of proteins, lipids, collagen, and bioactive compounds from seafood waste [55,57]. Compared with conventional extraction methods, UAE can reduce extraction time, solvent consumption, and energy requirements. In addition, the process can be integrated with enzymatic, thermal, or solvent-based methods to improve extraction performance. However, prolonged ultrasound treatment may lead to degradation or denaturation of sensitive biomolecules [36,55].

### 5.4. Microwave-Assisted Extraction (MAE)

Microwave-assisted extraction combines microwave heating with solvent extraction to accelerate heat and mass transfer within the sample matrix. Rapid internal heating significantly reduces extraction time and may improve recovery of target compounds compared with conventional extraction techniques. MAE has demonstrated promising applications in the extraction of lipids, proteins, and other bioactive compounds from seafood by-products. However, non-uniform heating and potential thermal degradation under severe conditions remain important limitations [58].

### 5.5. Cold Plasma

Cold plasma is a non-thermal technology based on partially ionized gases containing reactive species such as ions, electrons, radicals, and UV photons. These reactive species can disrupt cellular structures and facilitate the release of intracellular compounds while minimizing thermal damage to heat-sensitive biomolecules. Cold plasma has shown potential for extraction of antioxidants, phenolic compounds, and essential oils from biological materials. However, its application in seafood waste valorization remains limited, particularly for high-lipid feedstocks where plasma-induced oxidation may negatively affect product quality [57,59].

Among these emerging technologies, subcritical water processing has attracted particular attention due to its dual function as both a green solvent and hydrolysis medium. Unlike many extraction technologies that primarily facilitate compound release, SWE can simultaneously hydrolyze biomass and recover multiple value-added compounds without requiring organic solvents. Under elevated temperature and pressure conditions, the physicochemical properties of water can be modified to enhance solubility, mass transfer, and hydrolysis reactions, making the process highly versatile for seafood waste valorization. Furthermore, the ability to directly process wet biomass eliminates the need for energy-intensive drying steps commonly required in conventional extraction techniques. As shown in Table 7, emerging technologies differ in terms of target compounds, advantages, limitations, and environmental impacts. Owing to its tunable solvent properties, rapid reaction kinetics, and environmentally friendly nature, subcritical water technology has emerged as one of the most promising approaches for sustainable seafood waste valorization and is therefore discussed in greater detail in the following section.

## 6. Hydrothermal Treatments

Hydrothermal treatments refer to thermal processing technologies that use liquid water at elevated temperatures and pressures, often under subcritical or supercritical conditions, as the reaction medium. The nature of the process depends largely on the reaction temperature and can generally be categorized as hydrothermal carbonization (typically 100–250 °C), hydrothermal liquefaction (typically 250–374 °C), and hydrothermal gasification (above 374 °C). Although solid, liquid, and gaseous products are formed across the entire temperature range, the dominant product varies with temperature: solid products prevail at lower temperatures (carbonization), liquid products dominate at intermediate temperatures (liquefaction), and gaseous products become predominant at higher temperatures (gasification) [60,61].

Within the intermediate temperature range, water exists in the subcritical region (100–374 °C and up to 22.1 MPa), where it remains in the liquid state while exhibiting significantly altered physicochemical properties. These include a reduced dielectric constant and viscosity, and increased diffusivity, which enhance its ability to dissolve, hydrolyze, and extract a wide range of compounds from biomass [62]. As a result, subcritical water has been widely used as an effective medium for hydrothermal extraction of valuable compounds from biomass and other organic materials. Depending on the operating conditions and process objectives, SWE may involve extraction, hydrolysis, carbonization, or liquefaction pathways.

**Figure 2:**
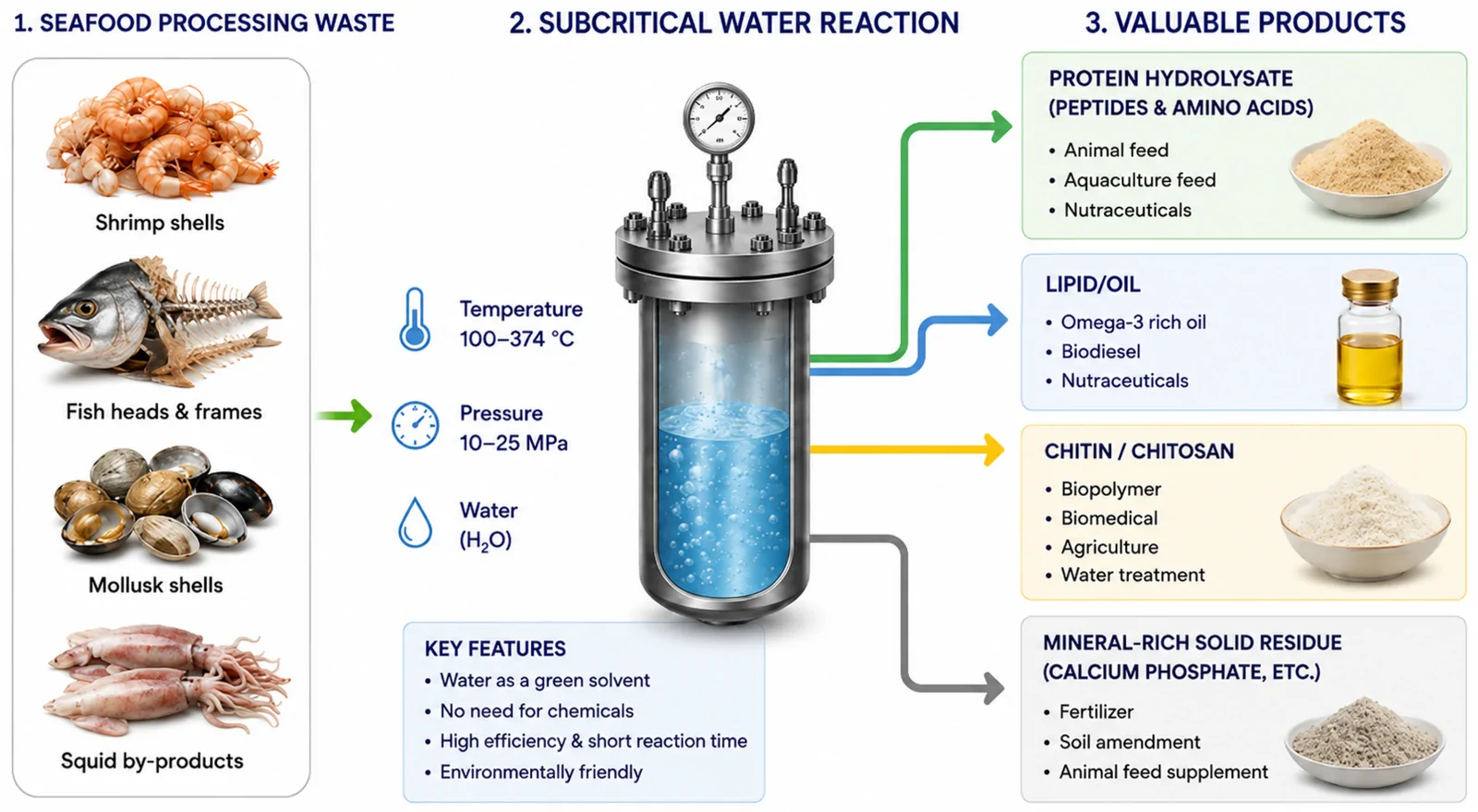
Conceptual overview of seafood processing waste valorization using SWE.

Subcritical water processes can produce a variety of value-added products, including peptides, amino acids, protein hydrolysates, omega-3-rich oils, free fatty acids, organic acids, oligochitosan, hydrochar, bio-oils, and mineral-rich residues (Figure 2). These products have potential applications in the food, nutraceutical, pharmaceutical, animal and aquafeed, cosmetic, biomedical, environmental, and bioenergy sectors. The quality and state of the final product are factors of the processing conditions and the initial composition of the feedstock.

Another major advantage of subcritical water processing is its operational flexibility. By adjusting parameters such as temperature, pressure, residence time, water-to-biomass ratio, and reaction atmosphere, the process can be directed toward selective extraction, hydrolysis, carbonization, or liquefaction pathways. Moderate temperatures generally favour the recovery of peptides and amino acids, whereas higher temperatures promote the formation of hydrochar, organic acids, and oil-rich fractions. This tunable nature makes subcritical water processing highly attractive for integrated biorefinery and circular bioeconomy applications.

SWE can also be integrated with other green technologies, such as supercritical CO_2_ extraction, enzymatic hydrolysis, and membrane separation, to improve overall process efficiency and product quality. The effectiveness of subcritical water processing is primarily attributed to the substantial changes in the physicochemical properties of water under these conditions, which are discussed in the following section.

### 6.1. Physiochemical Properties of Subcritical Water

**Figure 3:**
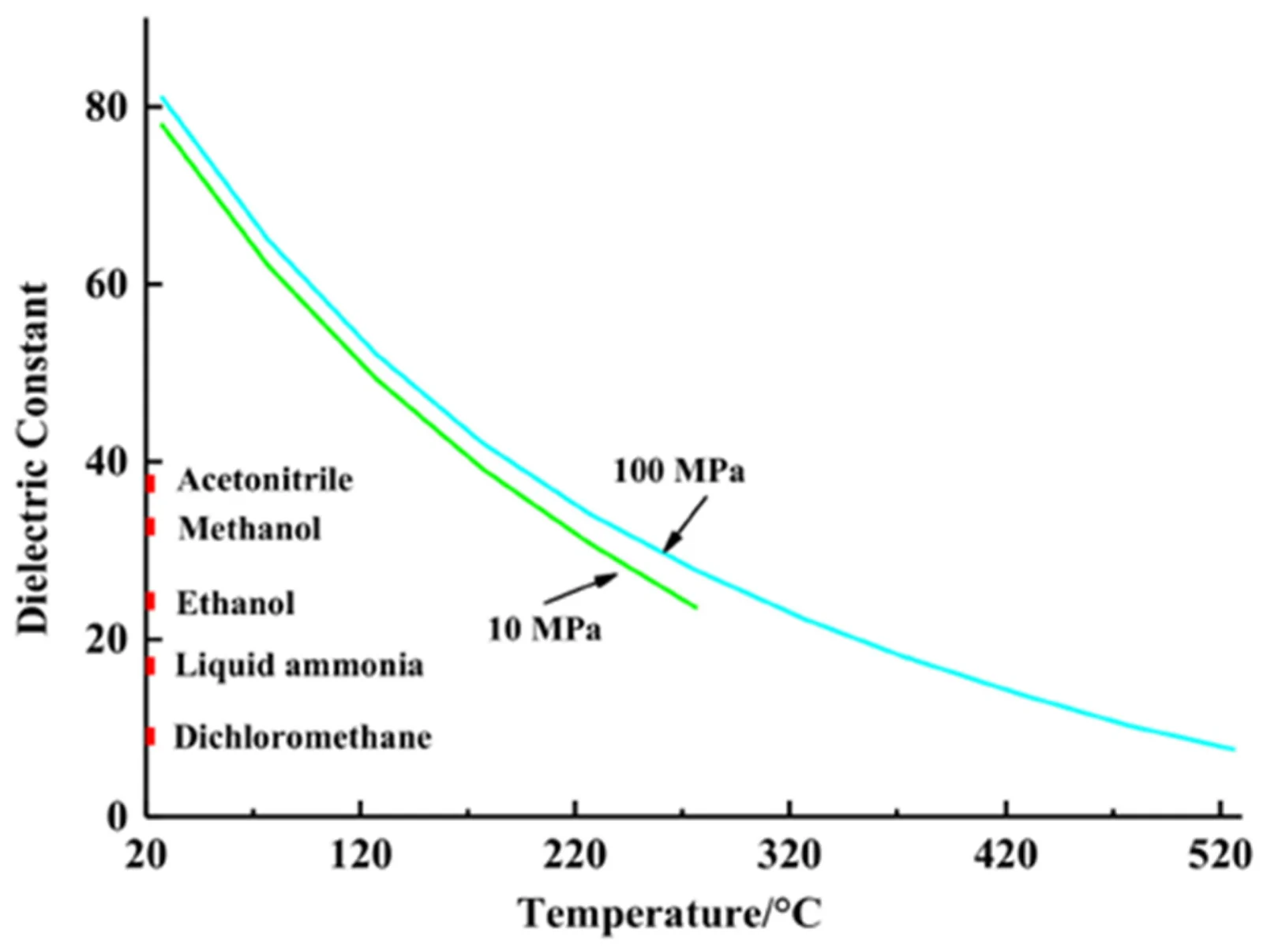
Changes in dielectric constant of water at different temperatures and pressures compared with selected organic solvents, reproduced from ref. [64].

Under subcritical conditions, water exhibits a unique combination of liquid-like density and gas-like diffusivity, and low viscosity, resulting in exceptional mass-transfer properties and making it an ideal solvent for extraction processes [63]. The relative permittivity (ε_r_), or dielectric constant (ε), of water decreases substantially from approximately 80 at ambient conditions to about 30 at 250 °C, and further to around 20 at 350 °C and 100 MPa due to the progressive disruption of the hydrogen bonds. As a result, water’s polarity decreases significantly, becoming equivalent to that of organic solvents such as acetonitrile, methanol, and ethanol (Figure 3) [64]. This tunable polarity enables the dissolution and extraction of moderately polar and low-polarity compounds that are otherwise poorly soluble in water under ambient conditions.

In addition, the viscosity and surface tension of water decrease substantially with increasing temperature, while its diffusivity increases, enhancing mass transfer and facilitating the penetration of water into the biomass matrix [65]. Under subcritical conditions, the significant increase in the ionic product of water ($K_{w}=\left[H^{+}\right]\left[OH^{-}\right]$) enhances the formation of hydronium (H_3_O^+^) and hydroxyl (OH^−^) ions, allowing water to act as both an acid and a base catalyst, and thereby promoting hydrolysis reactions without the need for external catalysts, acids, or alkalis [64].

These physicochemical changes allow subcritical water to function simultaneously as a solvent, reactant, and catalyst, enabling the selective recovery and transformation of valuable compounds from seafood processing by-products. Depending on the operating conditions, subcritical water can facilitate the extraction and production of amino acids, peptides, organic acids, lipids, collagen-derived products, carotenoids and other natural pigments, as well as mineral-rich residues, making it a versatile medium for biorefinery applications.

The unique physicochemical properties of subcritical water, such as enhanced ionization and decreased polarity, promote a series of hydrolysis, degradation, and transformation reactions within the major biochemical constituents of seafood processing by-products, including proteins, lipids, and carbohydrates. The extent and selectivity of these reactions depend primarily on temperature, residence time, pressure, and biomass composition.

### 6.2. Hydrolysis Mechanism of Proteins Under Subcritical Water Conditions

Under high-pressure and high-temperature conditions, the peptide bonds of the protein undergo hydrolysis reactions. This process is commonly described by an irreversible first-order reaction model. Hydronium and hydroxyl ions present in the medium act as catalysts, facilitating acid- and base-catalyzed peptide bond cleavage. In acid-catalyzed hydrolysis, protonation of the amide carbonyl oxygen facilitates nucleophilic attack by water molecules, resulting in bond cleavage and the formation of smaller peptides and free amino acids [66].

**Figure 4:**
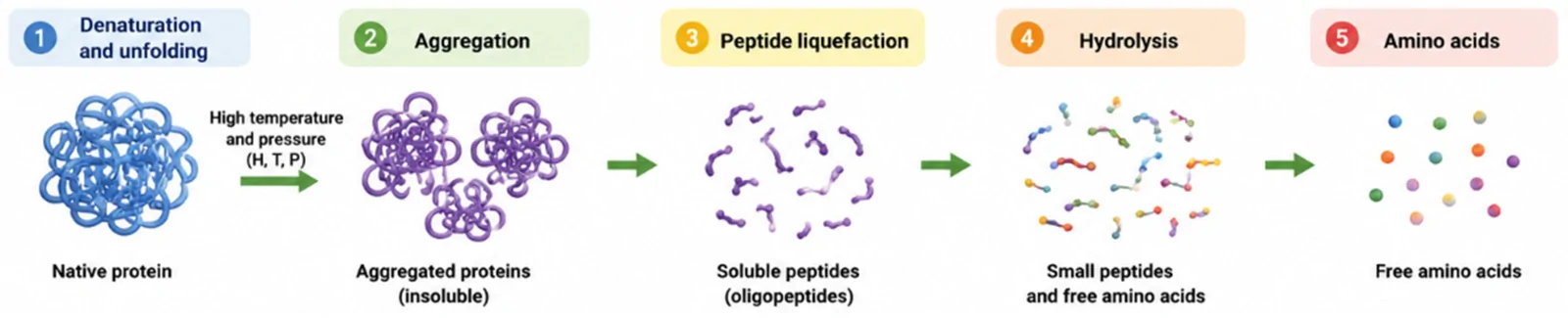
Protein hydrolysis mechanism in subcritical water. Blue structures represent native proteins; purple structures represent aggregated proteins and soluble peptides; multicolored chains represent small peptides; and colored spheres represent free amino acids. Green arrows indicate the progression of the hydrolysis process.

Under more extreme hydrothermal liquefaction conditions, proteins initially undergo denaturation, resulting in the loss of native secondary, tertiary, and quaternary structures due to disruption of hydrogen bonding and hydrophobic interactions. This unfolding increases solvent accessibility and exposes peptide bonds to hydrolytic attack. Subsequent hydrolysis leads to the formation of soluble peptides and free amino acids through catalytic cleavage in hot compressed water [66]. The overall hydrolysis process proceeds through a sequence of overlapping stages, outlined in Figure 4 below.

The high temperature and pressure conditions denature the native protein structure by disrupting weak intermolecular interactions such as hydrogen bonds and hydrophobic interactions. This results in the loss of higher-order structure and increased accessibility of peptide bonds to hydrolytic reactions [66]. The denatured and unfolded proteins often undergo a transient aggregation or gel formation stage. Protein aggregation can act as a barrier to the efficient production and purification of bioactive molecules and may negatively impact product quality and functionality. Aggregation may result from unfolding intermediates and unfolded states, protein self-association, chemical cross-linking, or partial chemical degradation. Mechanical agitation or mixing during the SWE, which is typically used to ensure homogeneity of the reaction mixture, can further enhance aggregation. In addition, elevated pressure and thermal conditions promote intermolecular interactions that facilitate aggregation. However, these aggregates are not necessarily stable; under continued subcritical water exposure, aggregated proteins can further dissociate and hydrolyze into smaller fragments such as polypeptides and amino acids over time [67]. Following aggregation, partial hydrolysis of peptide chains occurs, leading to the breakdown of large aggregates into soluble oligopeptides and intermediate molecular weight fragments. This stage represents a transition from insoluble protein assemblies to a homogeneous aqueous phase. The extent of liquefaction is strongly dependent on reaction temperature, residence time, and the severity of hydrothermal conditions. During this stage, hydrothermal conditions (high temperature and pressure) combined with the catalytic action of hydronium and hydroxyl ions begin to promote peptide bond activation. In peptide bonds, protonation at the amide nitrogen followed by nucleophilic attack leads to bond cleavage, initiating the conversion of aggregated proteins into soluble peptide fragments [66].

In the final stage, peptide bonds are progressively cleaved under subcritical water conditions, yielding low-molecular-weight peptides, free amino acids, and other small nitrogen-containing compounds. The hydrolysis reaction is promoted by the catalytic action of hydronium and hydroxyl ions generated in situ in hot compressed water. The extent of hydrolysis increases with reaction severity; however, excessive temperature or prolonged residence time may also lead to secondary degradation reactions of amino acids, reducing overall yield, altering product composition, and negatively impacting protein quality [66].

#### 6.2.1. Reaction Pathways of Amino Acids Under Subcritical Water Conditions

Once formed, amino acids undergo a network of competing degradation pathways under hydrothermal conditions. The dominant reactions are deamination and decarboxylation. Deamination leads to the formation of organic acids and ammonia (NH_3_), while decarboxylation produces amines and carbon dioxide (CO_2_). The relative contribution of these pathways depends on amino acid structure and reaction severity [68].

In addition, amino acids undergo side-chain-specific transformations. Hydrophobic amino acids typically follow deamination and decarboxylation routes, yielding small organic acids and nitrogenous compounds. Amino acids containing reactive functional groups may undergo dehydration, cleavage, or rearrangement reactions, while sulfur-containing amino acids form oxidized products such as sulfoxides and sulfonic acid derivatives [68].

These degradation products further participate in secondary reactions. Amino acids and amines can react with carbonyl-containing compounds derived from co-existing biomass components via Maillard-type reactions, forming nitrogen-containing heterocycles such as pyrroles, pyrazines, indoles, and pyridines. These compounds contribute significantly to nitrogen incorporation in the biocrude fraction [68].

Simultaneously, ammonia generated from deamination may react with fatty acids to form amides, while continued thermal degradation leads to gaseous products such as NH_3_ and CO_2_. Consequently, nitrogen is redistributed among the aqueous phase (organic acids and amines), gaseous phase (NH_3_ and CO_2_), and organic phase (heterocyclic compounds), depending on reaction severity and conditions [68].

#### 6.2.2. Side Reactions with Proteins, Peptides, and Amino Acids

Prolonged exposure or higher temperatures can lead to secondary degradation reactions, including deamination of amino acids and oxidation of specific side chains, such as the conversion of cysteine to sulfonic acid and methionine to sulfoxide. Under subcritical water conditions, pH is temperature-dependent, typically decreasing at lower temperatures (below ~180 °C) and increasing at higher temperatures (above ~200 °C), primarily due to amino acid degradation and the formation of Maillard reaction products. In addition, amino acid residues such as cysteine, methionine, and tryptophan are susceptible to oxidation. Furthermore, certain proteins (e.g., bovine serum albumin) exhibit limited disulfide bond cleavage, which can contribute to incomplete hydrolysis and increased product heterogeneity [66].

### 6.3. Hydrolysis Mechanism of Lipids Under Subcritical Water Conditions

**Figure 5:**
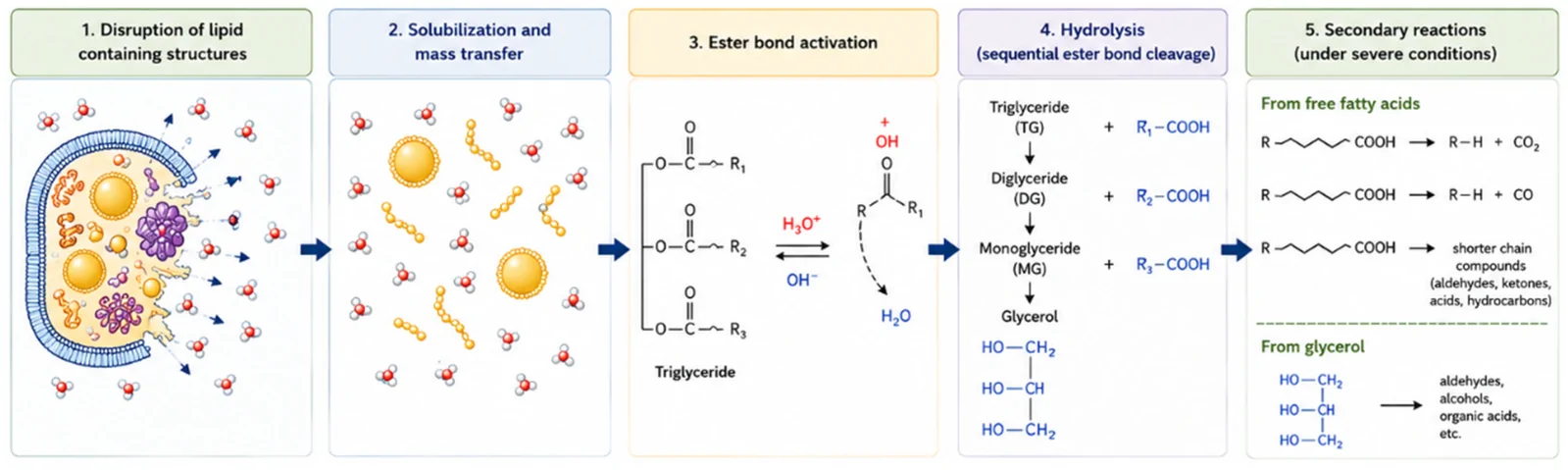
Lipid hydrolysis mechanism in subcritical water through the action of hydronium ions. Hydronium ions are represented by red and white spheres, lipid molecules by orange circles, fatty acid chains by orange chains, and proteins and other cellular components by purple structures. Blue arrows indicate process progression, and black arrows indicate the chemical reaction pathways.

Under subcritical water conditions, lipids undergo hydrolysis reactions that convert complex lipid molecules into free fatty acids, glycerol, and other low-molecular-weight compounds. The hydrolysis process is facilitated by the unique physicochemical properties of water at elevated temperature and pressure. These changes weaken intermolecular interactions such as hydrogen bonding, van der Waals forces, and hydrophobic interactions that bind lipids to cellular structures and tissue matrices. The stages of the lipid extraction process are illustrated in Figure 5.

The initial stage of extraction is governed by changes in the solvent properties of water. Under subcritical conditions, the reduction in dielectric constant increases the solvation capacity of water for hydrophobic compounds, while maintaining sufficient polarity to interact with more polar lipid species. This tunable solvent behavior allows selective extraction of different lipid classes. Lower temperatures (100–150 °C) generally favor the extraction of polar lipids such as phospholipids, as water remains relatively polar, whereas higher temperatures (200–300 °C) enhance the recovery of less polar lipid fractions, including triglycerides, due to the decreased polarity of water. The enhanced solvation capacity facilitates the diffusion of lipid molecules from the biomass matrix into the surrounding fluid [63]. Under subcritical conditions, rapid pressure fluctuations can generate microscopic vapor bubbles that subsequently collapse, producing localized shear forces and mechanical stress. This phenomenon, known as cavitation, contributes to the disruption of cellular membranes and tissue structures. The resulting increase in surface area and porosity enhances solvent penetration into the biomass matrix and improves mass transfer between lipid-containing regions and the extraction fluid [63].

Elevated temperatures promote the thermal degradation and hydrolysis of structural components associated with biological tissues. In fish waste, thermal treatment weakens cellular membranes, connective tissues, and lipid-protein complexes that encapsulate lipid droplets. The disruption of these structures increases the accessibility of intracellular lipids and facilitates their release into the extraction medium. Thermal hydrolysis also contributes to the breakdown of membrane-associated materials that may otherwise limit solvent penetration and lipid recovery [63].

Following structural disruption, the modified polarity of subcritical water promotes the solubilization of lipids. The solvent penetrates the disrupted tissue matrix and dissolves membrane lipids, phospholipids, and triglycerides. As solubilization proceeds, lipids are progressively transferred from intracellular compartments into the fluid phase. The combined effects of enhanced solvent properties, structural disruption, and improved mass transfer contribute to efficient lipid extraction [63]. Under severe hydrothermal conditions or prolonged residence times, extracted lipids may undergo secondary reactions. Triglycerides can be hydrolyzed to form free fatty acids and glycerol, while unsaturated fatty acids may experience oxidation, thermal cracking, cyclization, or other degradation reactions. These reactions can alter the composition and nutritional value of the recovered lipid fraction and influence the distribution of products among the oil, aqueous, and gaseous phases.

#### Reaction Pathways of Lipids Under Subcritical Water Conditions

Under hydrothermal liquefaction (HTL) conditions, lipids primarily undergo hydrolysis reactions that convert complex lipid molecules into free fatty acids (FFAs), glycerol, and intermediate glycerides. Triglycerides, which constitute the major lipid fraction in fish waste, are rapidly hydrolyzed through a series of reversible reactions involving diglyceride and monoglyceride intermediates. The first hydrolysis step, corresponding to the conversion of triglycerides to diglycerides, is generally considered the rate-limiting stage of the overall process. Lipid hydrolysis is catalyzed by the generated hydronium and hydroxyl ions under hydrothermal conditions and is strongly influenced by temperature, pressure, water-to-lipid ratio, and mass transfer limitations [68].

Once formed, glycerol and free fatty acids follow distinct reaction pathways. Glycerol is relatively unstable under hydrothermal conditions and undergoes a series of degradation, dehydration, and condensation reactions. These transformations produce a range of oxygenated intermediates, including aldehydes, ketones, alcohols, and organic acids. Representative products include formaldehyde, acetaldehyde, propionaldehyde, acrolein, methanol, ethanol, and allyl alcohol. Subsequent condensation reactions involving these intermediates contribute to the formation of bio-oil components [68].

In contrast, free fatty acids exhibit relatively high stability under subcritical water conditions but become increasingly reactive as reaction severity increases. Under more severe hydrothermal conditions, fatty acids can undergo decarboxylation and decarbonylation reactions, resulting in the formation of long-chain hydrocarbons. These hydrocarbons may subsequently undergo hydrogenation to produce alkanes or dehydrogenation to form alkenes. In addition, partial deoxygenation reactions can generate alcohols, which may react with free fatty acids through esterification pathways to form fatty acid esters [68].

Interactions between lipid-derived and protein-derived products also contribute significantly to product formation. Ammonia released during amino acid deamination can react with fatty acids to produce fatty acid amines and amides, thereby incorporating nitrogen into the organic phase. These reactions influence both the nitrogen content and chemical composition of the resulting biocrude.

Overall, lipid conversion during HTL can be described by the following generalized pathways:

1. Triglycerides → Diglycerides → Monoglycerides → Free fatty acids + Glycerol;
2. Glycerol → Aldehydes, alcohols, organic acids → Condensation products;
3. Free fatty acids → Hydrocarbons, alcohols, esters, amides.

Depending on reaction severity, these pathways result in the distribution of products among the oil, aqueous, gaseous, and, under certain conditions, solid phases.

### 6.4. Hydrolysis Mechanism of Carbohydrates (Chitin/Glycogen) Under Subcritical Water Conditions

Under subcritical water conditions, carbohydrates primarily undergo hydrolysis reactions through the cleavage of glycosidic bonds linking monosaccharide units. Hydrolysis is initiated by protonation of the glycosidic oxygen atom, which weakens the C–O bond and promotes bond cleavage. Subsequent nucleophilic attack by water molecules results in the formation of monosaccharides and shorter oligomeric fragments. The overall extent of hydrolysis is strongly influenced by temperature and residence time, while pressure generally has a lesser effect.

**Figure 6:**
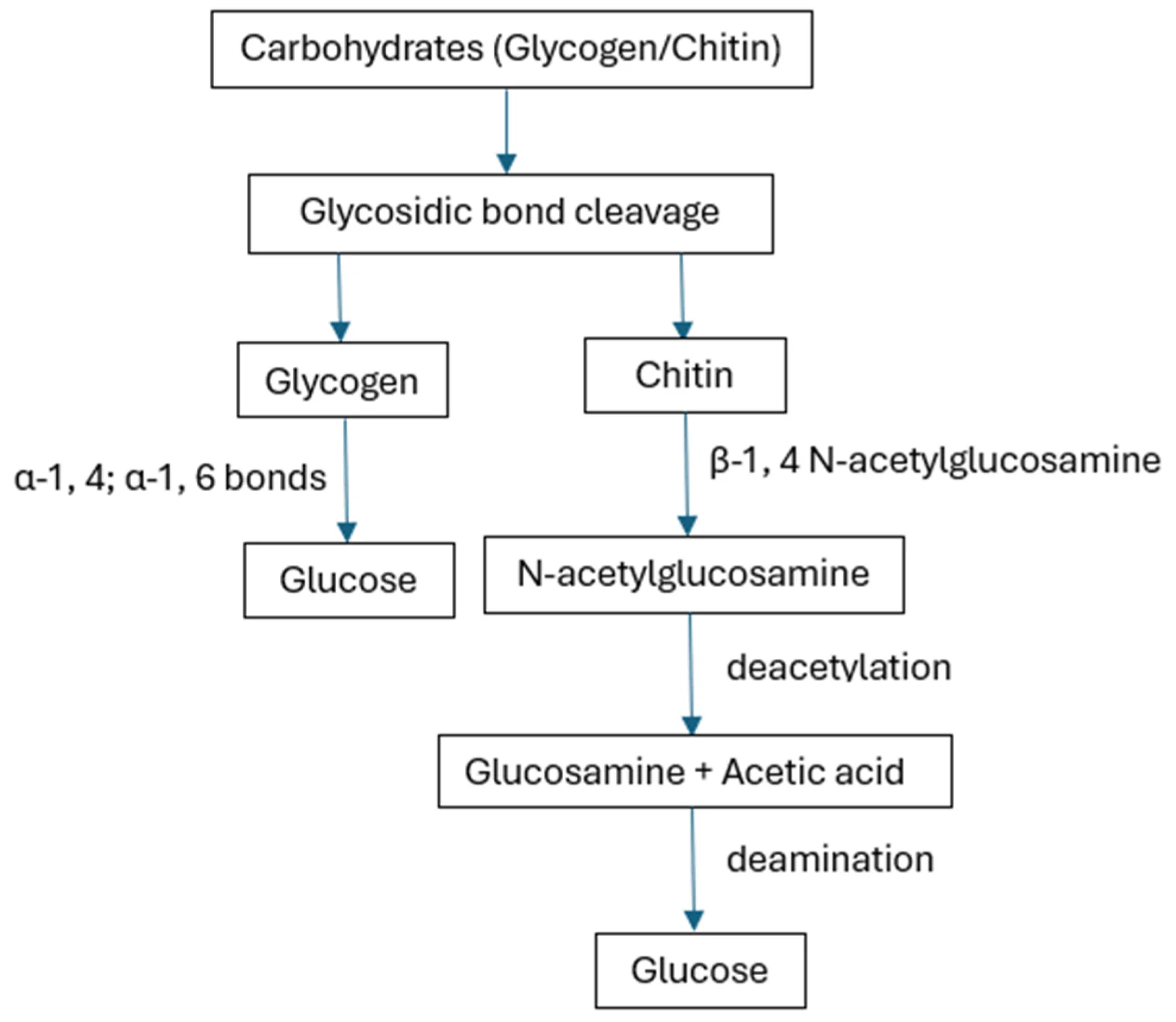
Schematic diagram of the carbohydrate hydrolysis under subcritical water conditions.

The hydrolysis behavior of carbohydrates under subcritical water depends significantly on their molecular structure, degree of crystallinity, and hydrogen-bonding network. Glycogen and chitin exhibit markedly different reactivities despite undergoing similar glycosidic bond cleavage mechanisms. Figure 6 shows the schematic diagram of the carbohydrate’s hydrolysis.

#### 6.4.1. Glycogen Hydrolysis

Glycogen is a highly branched, amorphous polysaccharide composed of glucose units connected through α-1,4 glycosidic bonds with α-1,6 branch points. Its relatively open structure, high water solubility, and limited crystallinity allow subcritical water to readily access glycosidic linkages, resulting in rapid hydrolysis compared with more crystalline polysaccharides such as chitin, and producing soluble oligosaccharides and glucose monomers [69,70].

As reaction severity increases, glucose undergoes a series of secondary reactions, including isomerization to fructose and mannose, dehydration to form 5-hydroxymethylfurfural (5-HMF), and fragmentation into low-molecular-weight organic acids such as lactic, acetic, and formic acids. Prolonged residence times may also promote condensation and polymerization reactions, resulting in the formation of insoluble carbonaceous products known as humins [71].

#### 6.4.2. Chitin Hydrolysis

Chitin is a crystalline, water-insoluble polysaccharide consisting of N-acetyl-D-glucosamine (NAG) units linked through β-1,4 glycosidic bonds. Although structurally similar to cellulose, chitin is significantly more resistant to hydrothermal degradation due to the presence of N-acetyl groups, which stabilize the crystalline structure through additional hydrogen bonding. As a result, chitin hydrolysis begins at approximately 280 °C, considerably higher than cellulose [72,73].

Hydrolysis initially occurs in the surface regions of chitin while the crystalline structure and NAG units remain largely intact up to around 320 °C. As temperature increases, hydrogen bonds and glycosidic linkages are progressively cleaved, leading to the destruction of the crystalline structure and release of NAG monomers and soluble oligomers. At temperatures approaching 360 °C, degradation extends into the interior of the polymer, resulting in extensive breakdown of the chitin matrix [72].

NAG undergoes almost complete deacetylation at temperatures above 170 °C, producing glucosamine and acetic acid. This reaction is primarily influenced by temperature and residence time, with pressure having a relatively minor effect [71]. The proposed mechanism involves protonation of the carbonyl oxygen of the N-acetyl group by hydronium ions, followed by nucleophilic attack by water and cleavage of the acetyl group. The resulting glucosamine subsequently undergoes deamination and rearrangement reactions, generating glucose and other sugar-derived intermediates [71].

#### 6.4.3. Reaction Pathways of Carbohydrates Under Subcritical Water Conditions

Following hydrolysis, carbohydrate-derived monosaccharides undergo a complex network of secondary reactions. Glucose and N-acetylglucosamine can isomerize to fructose and other sugar intermediates, which subsequently undergo dehydration reactions to form furans such as 5-HMF. Fragmentation and retro-aldol reactions produce low-molecular-weight compounds, including lactic acid, acetic acid, formic acid, and glyceric acid.

In chitin-containing systems, deacetylation generates acetic acid, while deamination produces ammonia and glucose-derived intermediates. These products may further participate in condensation and polymerization reactions leading to the formation of humins and other carbonaceous solids. Consequently, carbon from the original carbohydrate fraction is distributed among the aqueous phase (organic acids and sugars), solid phase (humins), and gaseous phase (CO_2_ and light gases), depending on reaction severity and residence time [71,72,73].

## 7. Practical Application of Hydrothermal Treatments

### 7.1. Factors Affecting the Performance of SWE

#### 7.1.1. Temperature

Temperature is the most influential parameter in subcritical water extraction and hydrothermal processing. Increasing temperature decreases the viscosity and surface tension of water while increasing diffusivity, thereby improving solvent penetration into the biomass matrix and enhancing mass transfer rates [74,75]. Elevated temperatures also weaken hydrogen bonds within the water structure, resulting in a lower dielectric constant and reduced polarity. Consequently, subcritical water becomes capable of dissolving moderately polar and non-polar compounds that are poorly soluble under ambient conditions [74,75].

In addition, increasing temperature enhances the ionic product of water, leading to higher concentrations of hydronium and hydroxyl ions that promote acid- and base-catalyzed hydrolysis reactions without the addition of external catalysts. As a result, higher temperatures generally improve the extraction and hydrolysis of proteins, lipids, and polysaccharides from seafood processing waste. However, excessive temperatures may promote secondary degradation reactions, including amino acid decomposition, oxidation of polyunsaturated fatty acids, and formation of Maillard reaction products. Therefore, temperature must be carefully optimized to maximize extraction efficiency while preserving the quality and functionality of recovered bioactive compounds [62].

#### 7.1.2. Extraction Time

Extraction time, hold time, or residence time is another critical parameter influencing the efficiency and selectivity of subcritical water processing. Short extraction times, typically ranging from 5 to 30 min, are commonly employed for the recovery of bioactive compounds from seafood by-products. Increasing extraction time generally enhances the extent of hydrolysis and extraction by allowing greater contact between the solvent and biomass [13,62].

Prolonged exposure to hydrothermal conditions may promote secondary degradation reactions, reducing the yield and functionality of target compounds. Extended residence times can lead to oxidation, polymerization, and condensation reactions, as well as the formation of free radicals that may alter the chemical structure and antioxidant properties of bioactive molecules. Consequently, an optimum extraction time should be selected to achieve high extraction yields while minimizing degradation of valuable products [62,74].

#### 7.1.3. Particle Size

Particle size significantly affects mass transfer and extraction efficiency during subcritical water processing. Reducing particle size increases the specific surface area available for solvent contact, shortens diffusion pathways, and facilitates the penetration of subcritical water into the biomass matrix. As a result, smaller particles generally enhance extraction rates and improve the recovery of proteins, lipids, and other bioactive compounds [62].

For this reason, grinding or milling is commonly employed as a pretreatment step prior to processing solid seafood residues such as fish frames, skin, bones, and crustacean shells. Nevertheless, excessively fine particles may increase slurry viscosity and create operational challenges, including reactor clogging and difficulties in downstream solid–liquid separation. Therefore, an appropriate particle size should be selected to balance extraction efficiency and process operability.

#### 7.1.4. Solid-to-Solvent Ratio

The solid-to-solvent ratio is an important factor affecting both extraction performance and process economics. A sufficient quantity of water is required to facilitate hydrolysis reactions, dissolve reaction products, and maintain efficient mass transfer between the biomass and the extraction medium. Increasing biomass loading can improve product concentration and reduce downstream processing costs. However, insufficient water availability may limit hydrolysis reactions, hinder mass transfer, and reduce extraction efficiency [13].

Low solvent volumes may promote secondary condensation and polymerization reactions due to the accumulation of reaction intermediates. Conversely, excessive water usage increases energy consumption associated with heating and product concentration. Therefore, an optimum solid-to-solvent ratio should be established for each feedstock and target product to achieve a balance between extraction efficiency and process economics [62,74].

#### 7.1.5. Pressure

The primary role of pressure in subcritical water processing is to maintain water in the liquid state at elevated temperatures and ensure intimate contact between the solvent and biomass. Typical subcritical water processes operate at pressures ranging from 50 to 100 bar, although higher pressures may be employed depending on operating temperature and reactor design [62].

Compared with temperature, pressure generally has a less pronounced effect on extraction efficiency. Nevertheless, pressure influences water density, diffusivity, and solvent properties, which may indirectly affect solubility, reaction kinetics, and mass transfer. Once sufficient pressure is applied to maintain liquid water conditions, further increases in pressure usually result in only minor improvements in extraction performance. Consequently, temperature is generally considered the dominant operating parameter in subcritical water processing, while pressure primarily serves a supporting role in maintaining the desired reaction environment [62,75].

### 7.2. Reactor Configurations for Subcritical Water Processing

Subcritical water extraction and hydrolysis processes are typically conducted in high-pressure reactors designed to maintain water in the liquid state at temperatures between 100 °C and 374 °C. Reactor configuration plays a critical role in determining heat and mass transfer characteristics, residence time distribution, extraction efficiency, product selectivity, and process scalability. Depending on the intended application, subcritical water processing can be carried out using batch, semi-batch, or continuous-flow reactor systems. Batch reactors are the most widely used configuration in laboratory-scale studies because of their simplicity and flexibility, whereas continuous-flow systems are increasingly being explored for industrial implementation due to their superior process control and scalability. The selection of an appropriate reactor design depends on factors such as feedstock characteristics, desired products, operating conditions, throughput requirements, and economic considerations.

#### 7.2.1. Batch Reactors

Batch reactors are the most commonly used reactor configuration for laboratory-scale subcritical water extraction and hydrolysis studies. A typical batch system consists of a high-pressure reactor vessel equipped with a heating system, thermal insulation, temperature and pressure monitoring devices, and safety features such as pressure relief valves. In a typical operation, the feedstock and water, with or without catalysts, are loaded into the reactor, which is then sealed and purged with an inert gas (e.g., nitrogen or argon) to remove residual air. The reactor is subsequently heated to the desired temperature and maintained for a predetermined residence time under autogenous or externally controlled pressure conditions. Upon completion of the reaction, the reactor is rapidly cooled to quench further reactions, and the products are recovered for subsequent analysis [64,76].

**Figure 7:**
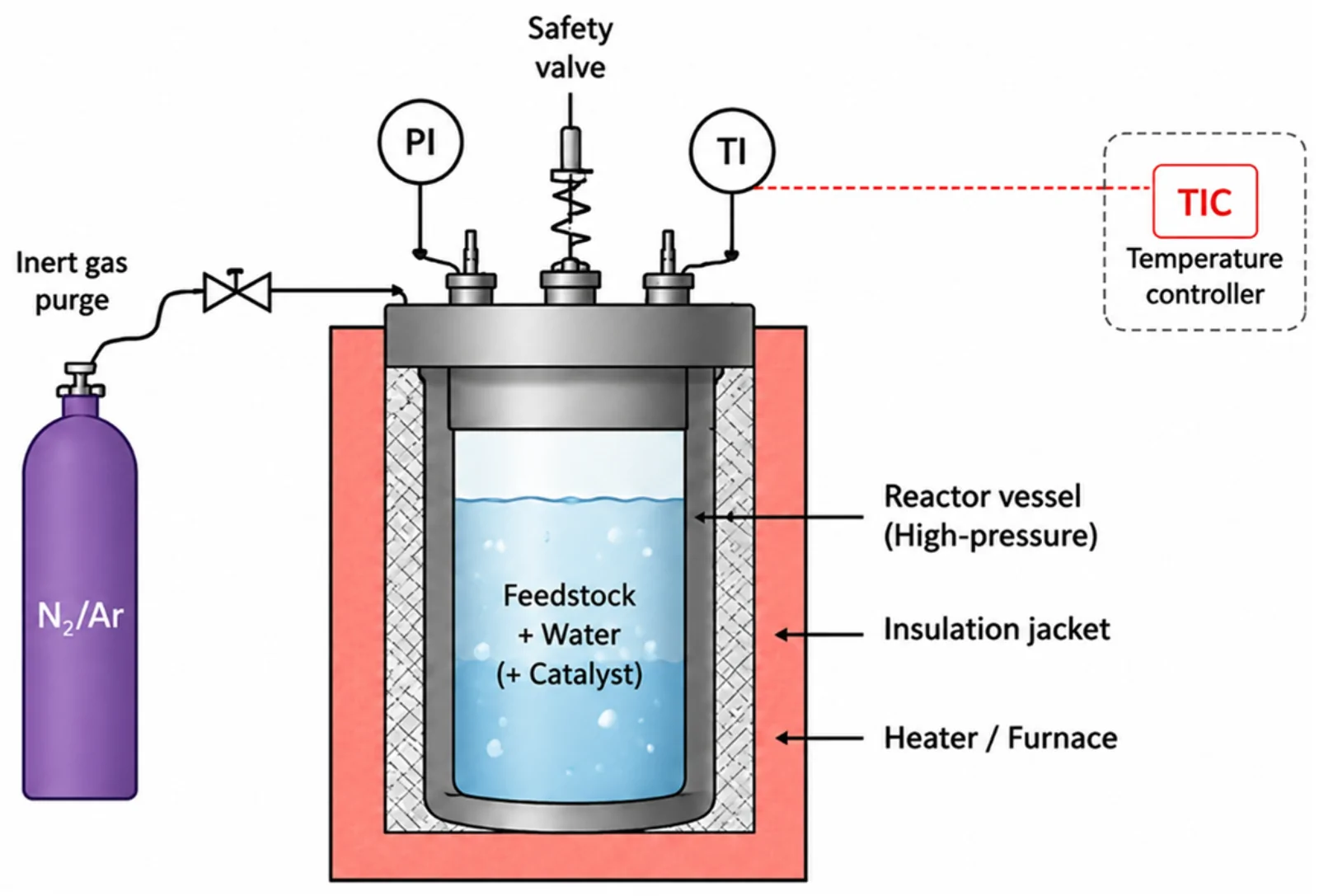
Schematic diagram of a typical batch reactor (dotted lines show the instrumentation and control signals).

Batch reactors offer several advantages, including simple design, low capital cost, operational flexibility, and suitability for evaluating the effects of processing variables. Consequently, they are widely employed for preliminary investigations and process optimization studies involving seafood waste valorization. However, batch systems are associated with relatively long heating and cooling periods, which can result in non-uniform temperature histories and broadened residence time distributions. As a result, reactions may occur during both the heating and cooling stages, making precise control of reaction severity and product selectivity more challenging [76]. Most studies on the SWE of fish and shellfish processing wastes have therefore been conducted using laboratory-scale batch reactors, particularly high-pressure stainless-steel autoclaves. Figure 7 presents a schematic illustration of a typical batch reactor used for subcritical water processing.

#### 7.2.2. Semi-Batch Reactors

In semi-batch reactor systems, the biomass feedstock is brought into contact with a continuous flow of the reaction medium, while only part of the inlet and/or outlet streams operate continuously. Unlike batch reactors, where both the feedstock and water are loaded into the reactor before heating, the biomass is typically placed in the extraction vessel and water is continuously pumped through the system once the target temperature and pressure have been reached. This configuration allows the extracted compounds and reaction products to be continuously removed from the reactor, thereby reducing their exposure to prolonged thermal treatment. Although some side reactions may occur, the main reactions take place only when the feedstock and water are both present and mixed with each other [76,77].

**Figure 8:**
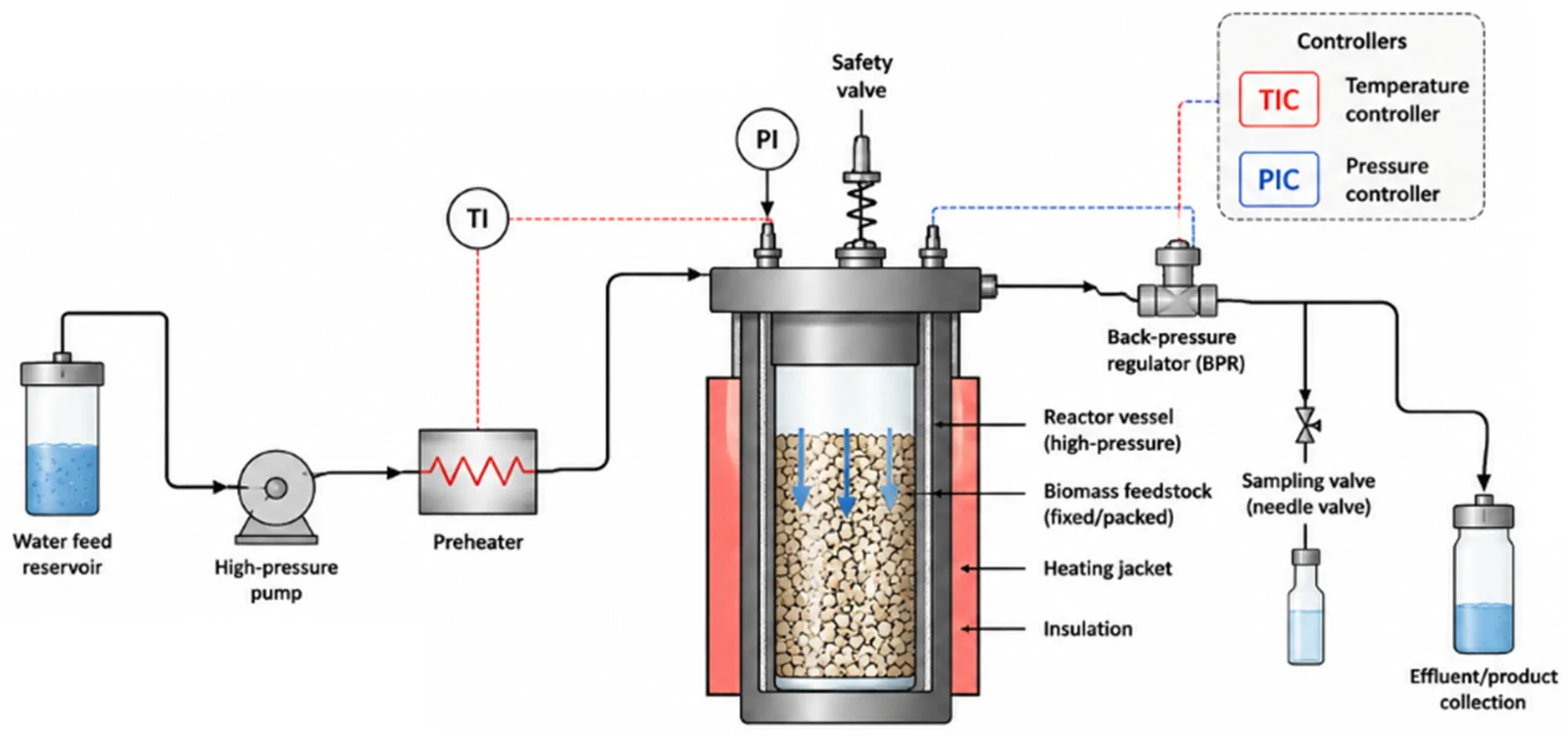
Schematic diagram of a semi-batch reactor system (dotted lines show the instrumentation and control signals).

The system pressure is maintained using a back-pressure regulator, while high-pressure pumps ensure a constant flow of water through the reactor. Product streams can be collected continuously or sampled periodically during operation, allowing monitoring of extraction and hydrolysis progress without interrupting the process [76]. Compared with batch systems, semi-batch reactors generally provide improved mass transfer, enhanced extraction efficiency, reduced secondary degradation of products, and easier solvent replacement. However, their operation is more complex and requires additional equipment, including high-pressure pumps, flow-control devices, and pressure regulation systems. Consequently, semi-batch reactors are particularly attractive for the recovery of thermally sensitive bioactive compounds and for studies requiring precise control of extraction conditions. Figure 8 presents a schematic illustration of a typical semi-batch reactor system.

#### 7.2.3. Continuous-Flow Reactors

In continuous-flow reactor systems, both the feedstock and water are continuously introduced into the reactor while products are continuously withdrawn. Unlike batch and semi-batch reactors, where the reaction time is determined by the duration of the experiment, the residence time in continuous systems is controlled by the reactor volume and the volumetric flow rate of the feed stream. This enables precise control of reaction severity and facilitates kinetic studies under well-defined operating conditions [76].

**Figure 9:**
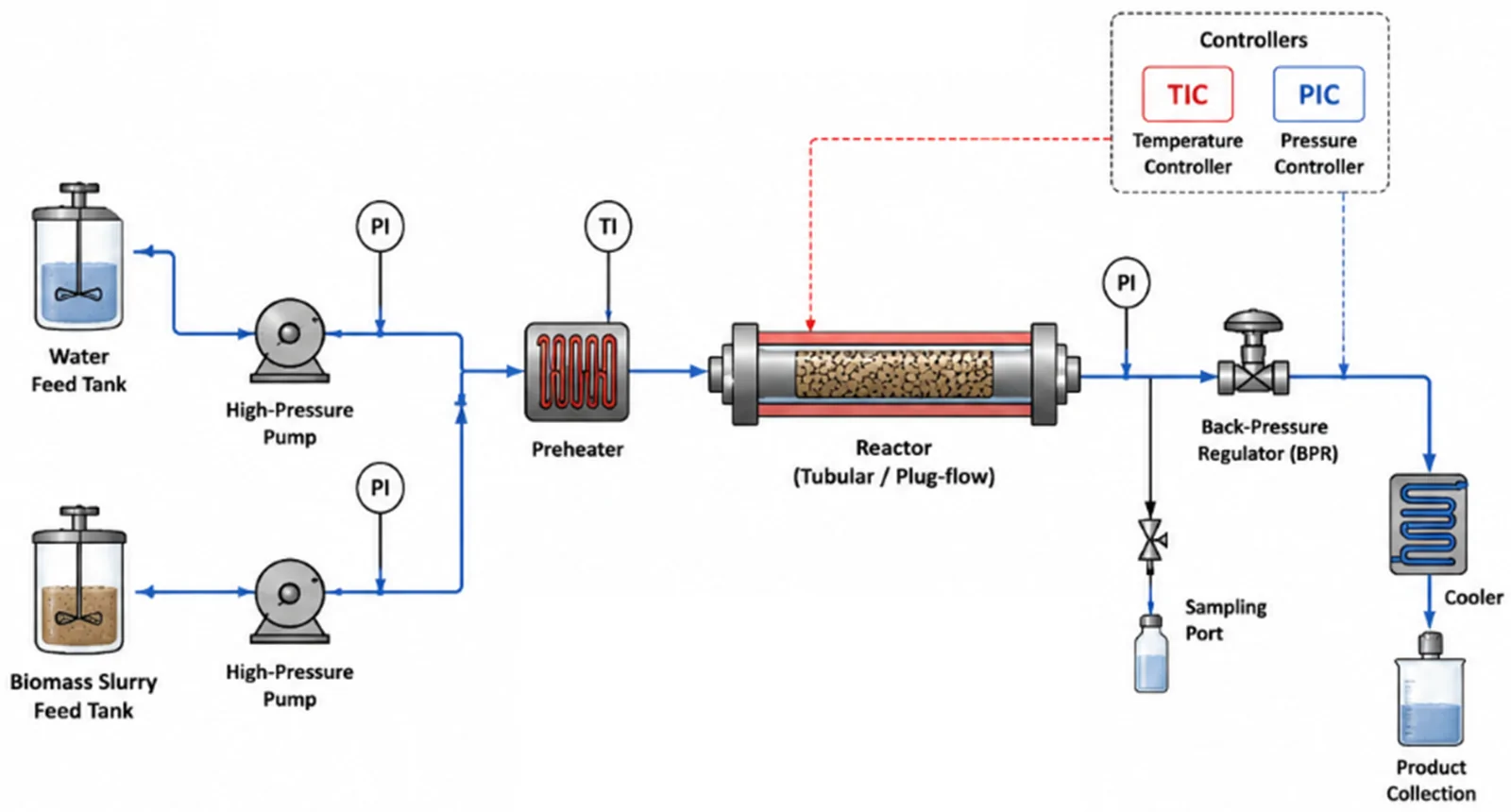
Schematic diagram of a continuous-flow reactor system (dotted lines show the instrumentation and control signals).

A typical continuous subcritical water processing system consists of feed tanks, high-pressure pumps, preheaters, a reactor section (e.g., tubular or plug-flow reactor), a back-pressure regulator, and product collection vessels. Such systems provide uniform operating conditions and allow rapid heating and cooling of the reaction medium, thereby minimizing undesirable secondary reactions. Consequently, continuous-flow reactors have been widely used to investigate reaction mechanisms and kinetics of model compounds as well as hydrothermal conversion pathways of biomass [76]. Figure 9 shows a schematic diagram of a continuous-flow reactor system.

Continuous operation offers several advantages, including precise residence time control, higher throughput, improved reproducibility, and greater potential for industrial-scale implementation. In addition, the continuous removal of products can reduce thermal degradation and improve product quality. However, continuous systems generally require higher capital investment and more sophisticated instrumentation than batch reactors. The handling and pumping of concentrated biomass slurries also present significant technical challenges, including equipment wear, flow instability, and reactor plugging caused by solid particles [64,77].

Although continuous hydrothermal processing has attracted considerable interest for commercial applications due to its potential for high carbon and energy recovery efficiencies, most laboratory-scale studies on seafood waste valorization have been conducted using batch or semi-batch reactors. Further research is therefore needed to address scale-up challenges and optimize continuous reactor designs for processing heterogeneous seafood residues. In some industrial applications, multiple semi-batch units operated in sequence may provide a practical alternative to fully continuous processing.

### 7.3. Seafood By-Product Valorization Using Subcritical Water

Subcritical water extraction is recognized as a green processing technology that can act as a tunable platform for the simultaneous recovery of multiple high-value streams such as proteins, amino acids, organic acids, lipids, and mineral-rich residues from fish waste, demonstrating its potential in integrated waste valorization processes and biorefinery approaches. The composition and functionality of the recovered fractions depend on the feedstock characteristics and processing conditions; however, the literature consistently demonstrates the ability of SWE to convert diverse seafood by-products into commercially valuable products suitable for food, feed, nutraceutical, pharmaceutical, biomedical, and bioenergy applications. To facilitate comparison across product classes, Table 8 summarizes the typical operating conditions, major products, reported yield ranges, selectivity under favorable conditions, and effects of excessive processing severity reported for the valorization of seafood by-products using subcritical water extraction.

#### 7.3.1. Amino Acids and Peptides

Amino acids and peptides are among the most extensively studied products recovered through SWE of seafood waste. Studies conducted within the temperature range of 180–260 °C and reaction times up to 60 min demonstrated the formation of a broad spectrum of amino acids from various marine wastes, including squid muscle, fish protein hydrolysate, tilapia scales, and blue mussels [10,79,80,81]. The yield of individual amino acids varied considerably among studies, reflecting differences in feedstock composition and processing conditions.

Increasing temperatures from 140 °C to 180 °C enhanced the release of free amino acids and promoted the production of low-molecular-weight peptides from tuna fish meal [78]. Similarly, the highest yields of essential amino acids and peptide fractions were reported near 170–180 °C, accompanied by increased antioxidant activity, reducing power, and lipid peroxidation inhibition [12]. However, several studies observed a decline in amino acid concentrations and antioxidant activity at more severe processing conditions, suggesting the occurrence of secondary degradation reactions [12].

Reaction atmosphere has also been shown to influence amino acid production. The use of CO_2_ instead of inert gases such as N_2_ enhanced hydrolysis efficiency through in situ acidification, resulting in increased yields of amino acids, including leucine, isoleucine, and histidine [65,78]. In some cases, SWE produced higher amino acid recoveries than conventional enzymatic hydrolysis while eliminating the need for externally added catalysts [78]. Collectively, these findings demonstrate that SWE can serve as an effective platform for the production of amino acid-rich and peptide-rich fractions from seafood processing waste.

#### 7.3.2. Protein Hydrolysate

Fish viscera, owing to their high protein content, have been identified as a promising feedstock for protein hydrolysate production through SWE. Studies conducted at temperatures between 140 and 220 °C and extraction times of approximately 5 min demonstrated efficient protein recovery, with optimal yields frequently reported around 180 °C [7]. Under controlled hydrothermal conditions (90–250 °C and 100 bar), near-complete recovery of the protein fraction was achieved, yielding extracts composed predominantly of collagen-derived peptides whose molecular weights decreased with increasing temperature [8].

The recovered protein hydrolysates exhibited a variety of desirable bioactive properties. For example, extracts obtained from fish viscera demonstrated significant anti-inflammatory activity without detectable cytotoxicity and remained stable under varying temperatures, pH conditions, and simulated gastrointestinal digestion, highlighting their potential applications in the food and nutraceutical industries [8,81]. Similarly, SWE of squid muscle produced extensive protein hydrolysis, together with hydrolysates exhibiting strong antioxidant activity and near-complete material conversion [79].

Several studies reported that hydrolysis severity enhanced the formation of low-molecular-weight peptides and free amino acids, which were closely associated with improved antioxidant and antimicrobial activities. Maximum bioactivities were commonly observed around 250–280 °C [6]. Interestingly, while hydrolysis efficiency generally increased with temperature, optimal antioxidant activity and essential amino acid content were often achieved at intermediate temperatures rather than under the harshest processing conditions, indicating that excessive degradation may compromise functional properties [12,79].

#### 7.3.3. Lipids and Organic Acids

In addition to protein-derived products, SWE can simultaneously generate valuable lipid-rich and organic-acid-rich fractions from seafood processing waste. Hydrothermal processing of fish meat and other marine residues promotes liquefaction of the biomass, resulting in the formation of an aqueous phase containing amino acids and organic acids, together with an oil-containing phase rich in lipids [65].

Several studies have reported the production of organic acids such as lactic acid and pyroglutamic acid during SWE of fish waste. These compounds remained relatively stable under hydrothermal conditions (temperatures between 240 °C and 280 °C and longer reaction times) and accumulated in the aqueous fraction, contributing to the overall value of the recovered products [65]. The formation of such compounds reflects the complex reaction pathways occurring during the decomposition of proteins and other organic constituents in seafood biomass.

SWE has also demonstrated considerable potential for lipid recovery from marine byproducts. Hydrothermal treatment promotes the hydrolysis of triglycerides into free fatty acids, facilitating the release and separation of lipid fractions from the biomass matrix [65,82]. Recovered oils have been shown to contain nutritionally valuable omega-3 polyunsaturated fatty acids, particularly eicosapentaenoic acid (EPA) and docosahexaenoic acid (DHA), particularly around 240 °C. However, the stability of these highly unsaturated fatty acids remains a challenge, as excessive processing severity may promote oxidation and thermal degradation. Therefore, balancing lipid recovery with the preservation of fatty acid quality remains an important consideration for industrial applications [82].

Beyond food and nutraceutical uses, the recovered lipid fractions may serve as feedstocks for biofuel production. The conversion of free fatty acids into fatty acid methyl esters has demonstrated the potential for integrating SWE into biorefinery platforms that simultaneously produce high-value bioactive compounds and renewable energy products from seafood processing residues [82].

#### 7.3.4. Biopolymers and Mineral-Rich Products

Beyond the recovery of soluble bioactive compounds, SWE has demonstrated significant potential for the extraction, purification, and structural modification of valuable biopolymers from seafood processing waste. Crustacean shells, fish skin, bones, and scales are particularly attractive feedstocks because they contain substantial quantities of chitin, collagen, and mineral components that can be transformed into value-added products.

One notable application of SWE is the recovery of chitin from crustacean shell waste. Effective deproteinization and recovery of high-purity α-chitin have been reported at around 260 °C using short reaction times, reducing the need for conventional chemical-intensive extraction methods [11]. In addition to removing proteins, SWE induced structural modifications in the recovered chitin, including increased crystallinity and alterations in the associated mineral phases such as calcite and hydroxyapatite. Such changes may enhance the suitability of the material for biomedical and advanced material applications [11].

Collagen-rich seafood by-products have also been successfully valorized using subcritical water technologies. Pepsin-solubilized collagen (PSC) recovered from mackerel skin and bones was subsequently hydrolyzed under subcritical water conditions at 200–250 °C to produce low-molecular-weight peptide fractions enriched in glycine and other bioactive amino acids [87]. These hydrolysates exhibited enhanced antioxidant activity and showed potential as functional ingredients for food, cosmetic, pharmaceutical, and nutraceutical applications [87].

In addition to biopolymer recovery, SWE generates mineral-rich solid residues that can serve as valuable co-products. Following the extraction of proteins and other organic constituents, the remaining solids are often enriched in calcium phosphate minerals, particularly hydroxyapatite [8]. These materials have attracted considerable interest for biomedical applications due to their similarity to the mineral component of natural bone. Potential applications include bone graft materials, tissue engineering scaffolds, dental materials, and other biomaterial products.

The simultaneous recovery of chitin, collagen-derived products, and mineral-rich residues highlights the versatility of SWE as an integrated biorefinery technology. By enabling the valorization of both organic and inorganic fractions of seafood waste, SWE contributes to improved resource efficiency and supports the development of circular bioeconomy approaches within the seafood processing sector.

## 8. Integrated Biorefinery Approaches for Seafood Waste Valorization

### 8.1. Conversion of Fish Waste into Bioenergy and Biochemicals

Subcritical water-based processes not only enable the extraction of multiple high-value fractions from waste materials but also facilitate their conversion into bioenergy carriers and biochemicals. In particular, hydrothermal conversion technologies such as hydrothermal carbonization (HTC) and hydrothermal liquefaction (HTL) provide efficient routes for transforming wet biomass residues into value-added products, including hydrochar, bio-oil (biocrude), and functional carbon materials, while simultaneously reducing the environmental burden associated with conventional waste disposal.

Hydrochar is a carbon-rich solid material produced via hydrothermal carbonization at temperatures typically ranging from 180 to 250 °C. It is characterized by a partially carbonized structure enriched with oxygen-containing functional groups and a developing porous morphology, which makes it suitable for applications such as solid fuel production, pollutant adsorption, and soil amendment [88]. The physicochemical properties of hydrochar are strongly dependent on feedstock composition and processing conditions, including dehydration, decarboxylation, and aromatization reactions occurring under hydrothermal conditions. Beyond energy applications, hydrochar can also be engineered into high-performance functional carbon-based materials. For instance, shrimp processing waste, particularly shrimp shells, has been successfully converted into hydrochar via HTC. The resulting material exhibited enhanced surface properties, including increased surface area and nitrogen-containing functional groups, which contributed to high adsorption capacity for contaminants such as anionic dyes [89].

Fish processing residues have also been investigated under both conventional hydrothermal carbonization (CHTC) and microwave-assisted hydrothermal carbonization (MHTC). In conventional HTC, treatment at 180 °C for 120 min yielded hydrochar with up to 35% solid recovery and physicochemical characteristics comparable to those obtained via microwave-assisted processing. In contrast, MHTC operates through volumetric microwave heating, enabling significantly faster heating rates and thus substantially reducing reaction time, though at slightly higher temperatures (approximately 200 °C) [90]. Overall, both approaches produce hydrochar with similar functional characteristics, although MHTC offers advantages in process intensification and energy efficiency.

In addition to solid carbon products, seafood waste can be effectively converted into liquid biofuels via hydrothermal liquefaction. HTL produces a complex liquid product known as bio-oil or biocrude under subcritical or near-critical water conditions. This bio-oil consists of a wide range of oxygenated and nitrogen-containing compounds, including fatty acids, phenolics, alcohols, esters, ketones, and amines, and can serve as a precursor for renewable transportation fuels following appropriate upgrading. Compared with conventional thermochemical conversion routes, HTL is particularly advantageous for wet biomass such as seafood waste, as it eliminates the need for energy-intensive drying and operates under relatively moderate temperatures.

Several studies have demonstrated the conversion of seafood residues into biocrude under HTL conditions. For example, small yellow croaker waste has been processed at temperatures between 200 and 250 °C, producing biocrude with improved fuel properties as temperature increased [91]. Specifically, higher temperatures reduced density and acidity while altering chemical composition, as confirmed by GC–MS analysis showing a decrease in fatty acids and an increase in aromatic compounds. However, such changes also reflect secondary reactions such as cracking and repolymerization, which can influence product distribution and quality. Optimal conditions around 300 °C and approximately 125 min residence time yielded biocrude with improved viscosity and overall fuel characteristics, indicating that both temperature and reaction time are critical parameters for tuning product properties [91].

Similarly, HTL of anglerfish waste has been shown to generate biocrude containing alcohols, phenols, acids, esters, ketones, and amines. Although the resulting oil exhibits relatively high density, viscosity, and acid value compared with conventional petroleum fuels, its properties are consistent with other biomass-derived oils and therefore suitable for further upgrading. Temperature plays a dominant role in determining yield and composition, with optimal bio-oil production observed around 250 °C. Furthermore, the addition of acetic acid (CH_3_COOH) has been reported to enhance bio-oil yield while reducing solid residue formation, likely by influencing reaction pathways and improving biomass solubilization under hydrothermal conditions [92].

### 8.2. Combination of Other Methods and SWE

Integrated green processing strategies have demonstrated strong potential for the complete valorization of fish-processing by-products through sequential fractionation and conversion of different biomass fractions. In particular, the integration of subcritical water systems with complementary green technologies enables stepwise recovery of lipids, proteins, peptides, and bioactive compounds, improving both resource efficiency and product selectivity in marine biorefinery systems.

A widely studied integrated approach combines supercritical CO_2_ (SC-CO_2_) extraction with subsequent subcritical water hydrolysis. In this sequential process, SC-CO_2_ selectively extracts non-polar lipid fractions, producing oils enriched in polyunsaturated fatty acids (PUFAs), particularly omega-3 fatty acids, as well as fat-soluble vitamins (A, D, E, and K) and lipophilic carotenoids such as astaxanthin. Compared with conventional solvent extraction, SC-CO_2_ also reduces co-extraction of undesirable contaminants, including heavy metals, thereby improving oil purity and nutritional quality. Following lipid removal, the defatted biomass is subjected to SWE hydrolysis, where proteins are depolymerized into peptides and free amino acids under hydrothermal conditions. The yield and composition of hydrolysates strongly depend on temperature, typically in the range of 160–280 °C, where increasing severity enhances protein breakdown but may also promote secondary degradation reactions.

This sequential SC-CO_2_/SWE strategy has been demonstrated in several marine waste systems. For example, extraction of yellow corvina head and viscera produced PUFA-rich oil via SC-CO_2_, followed by SWE of the oil-free biomass between 160 and 235 °C. The maximum free amino acid yield was obtained at 235 °C, with the resulting hydrolysates showing significant bioactivities, including antioxidant, antidiabetic, and anticancer properties [83]. Similarly, sardine processing waste has been valorized using SC-CO_2_ extraction, recovering omega-3-rich fish oil containing up to ~17% PUFAs, followed by SWE yielding peptide- and amino acid-rich protein hydrolysates [85]. Pre-treatment of the feedstock by defatting was shown to enhance protein accessibility and improve hydrolysis efficiency, highlighting the importance of process sequencing in integrated systems. In addition, conger eel by-products have been processed using the same combined approach, producing edible oils and amino acid-rich fractions. In this case, SWE of the defatted biomass at 160–280 °C resulted in increasing amino acid release, while the highest antioxidant activity was observed at 280 °C [84], indicating that optimal conditions may differ depending on whether yield or bioactivity is targeted.

Beyond purely physicochemical fractionation, enzymatic hydrolysis has also been effectively integrated with hydrothermal pretreatment for fish by-product valorization. In one study, hydrothermal pretreatment of tilapia scales at 135 °C for 90 min significantly enhanced protein recovery (84.81%) without requiring prior demineralization. This pretreatment disrupted the mineralized collagen matrix, improving substrate accessibility and increasing the degree of hydrolysis during subsequent Alcalase enzyme treatment. The resulting gelatin-derived hydrolysates consisted mainly of low-molecular-weight peptides and exhibited strong angiotensin I-converting enzyme (ACE) inhibitory activity. Importantly, the bioactivity of these hydrolysates remained stable under thermal treatment, pH variations, and gastrointestinal digestion conditions, demonstrating their potential as functional food ingredients [81]. This highlights the synergistic effect of combining hydrothermal processing with enzymatic hydrolysis to improve both yield and biofunctionality.

Another promising integrated pathway involves coupling hydrothermal carbonization (HTC) with downstream extraction and fractionation strategies. HTC of fish and shrimp wastes under both conventional and microwave-assisted heating produces a solid hydrochar phase along with a process liquid (biocrude liquor) containing a wide range of valuable compounds. The hydrochar provides a carbon-rich solid matrix, while the liquid fraction contains oxygenated and nitrogen-containing compounds, including diketopiperazines, pyrazines, fatty acids, and sterols, depending on feedstock composition and reaction conditions. Microwave-assisted HTC has been shown to alter reaction pathways due to rapid volumetric heating, often increasing the relative abundance of nitrogen-containing heterocyclic compounds such as pyrazines compared with conventional HTC. This demonstrates that not only feedstock composition but also heating mode significantly influences product distribution and chemical selectivity [93].

A summary of representative studies investigating seafood waste valorization using SWE is presented in Table 9.

## 9. Challenges and Future Perspective

The diverse physical and chemical characteristics of seafood processing wastes make their management challenging. Variations in feedstock quality, processing efficiency, and the environmental impacts of different treatment methods are among the major challenges associated with seafood waste management [94]. Generally, thermochemical processes are preferred over biochemical processes in practical applications, due to the longer processing times, lower conversion efficiencies, and higher pretreatment costs associated with biochemical technologies. SWE is a promising thermochemical process that offers the distinct advantage of producing multiple value-added products and enabling the full valorization of waste streams, compared to the single-product outcomes typically obtained from biochemical processes [94]. In addition, no costly drying pretreatment is required, since SWE is particularly suitable for high-moisture biomass commonly found in seafood processing by-products.

Although SWE has shown significant potential for the recovery of high-value compounds from seafood wastes, several bottlenecks continue to hinder its industrial implementation and shape current research directions. One of the major challenges is the high capital and operational cost associated with high-pressure processing equipment. Integrating SWE with other green technologies, such as ultrasound-assisted extraction, microwave-assisted extraction, or supercritical CO_2_ extraction, may help reduce operating temperatures and energy consumption while maximizing resource recovery through an integrated biorefinery approach.

In addition, scaling up the process from laboratory-scale batch operation to industrial-scale continuous processing remains challenging because slurry pumpability is limited under high-pressure conditions. These limitations primarily arise from restrictions on the solid loading and particle size that pumping systems can effectively handle at elevated pressures. Therefore, further research is needed to develop pumping systems capable of handling high-solids slurries under high-pressure conditions, along with improved biomass pretreatment and slurry preparation techniques [95].

The accumulation of solid particles in the product stream represents another major operational challenge, as it can lead to clogging of the reactor outlet and downstream processing equipment. Consequently, careful optimization of operating conditions is required to minimize solid residue formation and improve product recovery. In laboratory-scale batch studies, product separation is commonly performed using solvents or physical methods such as filtration and centrifugation; however, these approaches may become economically impractical for large-scale industrial applications [95]. Therefore, the development of continuous and energy-efficient separation technologies is essential for industrial-scale operations. Potential solutions include the integration of continuous solid–liquid separation systems such as hydrocyclones, decanter centrifuges, membrane filtration, screw presses, and settling units. These technologies may improve separation efficiency while reducing operational costs and processing times.

Furthermore, several advancements are still required to improve process efficiency and facilitate large-scale implementation. Careful optimization of process parameters, including temperature, residence time, and solid loading, is essential to maximize product yields while preserving product quality and preventing thermal degradation [13]. Scaling up the process also requires improvements in equipment design, process automation, and economically feasible implementation strategies. More comprehensive studies using a wider range of fish processing by-products are needed to further evaluate the sustainability and economic viability of SWE processes compared to conventional solvent-based extraction methods in terms of extraction yield, product purity, and overall cost-effectiveness. In addition, meeting regulatory requirements for the use of SWE-derived products in food, pharmaceutical, and cosmetic applications remains essential for broader industrial adoption of this technology [13].

Addressing these challenges through continued research and technological innovation will support the full utilization of SWE as a sustainable and efficient approach for the valorization of seafood processing waste streams.

## 10. Conclusions

Seafood processing industries generate large quantities of by-products that are often underutilized despite their high content of valuable biomolecules such as proteins, lipids, minerals, and polysaccharides. The effective valorization of these residues is essential for reducing environmental impacts, improving resource efficiency, and supporting the transition toward a circular bioeconomy within the seafood sector. Conventional extraction and processing techniques have played an important role in recovering useful compounds from seafood processing wastes. However, their reliance on harsh chemicals, high energy consumption, and multi-step processing has highlighted the need for more sustainable alternatives.

SWE has emerged as a promising green technology capable of simultaneously extracting, hydrolyzing, and converting multiple components of seafood biomass into value-added products. The unique physicochemical properties of water under subcritical conditions enable it to function as a solvent, reactant, and catalyst, facilitating the breakdown of complex biomolecules and the recovery of peptides, amino acids, lipid fractions, organic acids, chitin derivatives, and mineral-rich materials. In addition, hydrothermal conversion pathways such as hydrothermal carbonization and hydrothermal liquefaction allow the transformation of seafood residues into bioenergy carriers and functional carbon materials. The versatility of SWE, together with its compatibility with wet biomass and reduced reliance on organic solvents, makes it particularly attractive for sustainable waste management and biorefinery applications.

Recent studies demonstrate that integrating SWE with complementary technologies, including supercritical CO_2_ extraction, enzymatic hydrolysis, and microwave-assisted processing, can further enhance resource recovery and enable stepwise fractionation of seafood by-products. Such integrated processing strategies offer promising routes for the development of marine biorefineries capable of producing multiple high-value products from a single feedstock. However, the full industrial potential of these technologies has yet to be fully realized. High capital costs associated with high-pressure reactors, difficulties in handling high-solid slurries, reactor clogging, and the need for efficient downstream separation technologies continue to limit commercial adoption. Further research is therefore required to optimize operating conditions, develop robust continuous-flow systems, and improve economic feasibility through process integration and intensification. In addition, comprehensive life cycle assessments (LCA) and techno-economic analyses (TEA) will be essential to evaluate the sustainability and competitiveness of SWE-based processes compared with conventional technologies.

Overall, SWE represents a versatile and environmentally friendly platform for the valorization of seafood processing wastes. Continued advancements in reactor design, process integration, and product separation technologies will play a crucial role in unlocking the full potential of this technology and supporting the development of sustainable marine biorefineries and circular resource management strategies in the seafood industry.

## Figures and Tables

**Table 1:** Regional overview of the landings and production, 2024 ^1^.

	Pacific	Inland	Atlantic	Canada
Commercial sea and freshwater fisheries ^2^				
Number of registered fishing vessels ^3^	1932	150	13,991	16,073
Total weight of landings (metric tonnes)	81,854	23,160	546,098	627,952
Total value of landings (in thousands of dollars)	385,230	84,005	3,627,498	4,012,728
Aquaculture ^4^				
Number of aquaculture establishments ^5^	170	154	291	615
Total weight of production (metric tonnes)	64,154	9734	86,430	160,318
Total value of production (in thousands of dollars)	562,814	60,918	741,126	1,364,858

^1^ Totals may not add up due to rounding. ^2^ Atlantic: Newfoundland and Labrador, Prince Edward Island, Nova Scotia, New Brunswick, Quebec; Inland: Ontario, Manitoba, Saskatchewan, Alberta, Northwest Territories and Nunavut; Pacific: British Columbia and Yukon. ^3^ Inland vessel count is for Ontario Great Lakes only. ^4^ Inland aquaculture totals include Ontario, Quebec, Territories, and suppressed values for Manitoba, Saskatchewan, and Alberta. ^5^ Source: Innovation, Science and Economic Development Canada, Canadian Industry Statistics (Aquaculture).

**Table 2:** Commercial sea fisheries landings by species groups and region, 2024 [15].

	Weight (Metric Tonnes)		
	Atlantic	Pacific	Canada
Groundfish	72,436	51,568	124,004
Pelagics	82,111	18,609	100,721
Shellfish	376,821	11,676	388,497
Other types of fish	13,003	0	13,003
Total	544,371	81,854	626,225

**Table 3:** Average composition of finfish by-products (by wet weight) and the commercially relevant constituents from each by-product type.

By-Product Type	Approximate Percentage of Whole Fish Weight (%)	Major Components
Head	10–25	Protein, lipids, minerals
Bones	10–15	Collagen, calcium, phosphorous, hydroxyapatite
Viscera (internal organs)	5–18	Protein, enzymes, lipids
Skin	3–6	Collagen/gelatin, bioactive peptides
Scales	~2	Collagen, hydroxyapatite
Swim bladder	Variable	Collagen/gelatin

**Table 4:** Representative composition of mixed seafood processing discards (dry matter basis).

Nutrient	Amount
Compounds:	
Crude protein	57.9 ± 5.3 (wt%)
Fat	19.1 ± 6.1 (wt%)
Ash	21.8 ± 3.5 (wt%)
Crude fibre	1.2 ± 1.2 (wt%)
Minerals:	
Calcium	5.8 ± 1.4 (wt%)
Phosphorous	2.0 ± 0.6 (wt%)
Potassium	0.7 ± 0.1 (wt%)
Sodium	0.6 ± 0.1 (wt%)
Magnesium	0.2 ± 0.0 (wt%)
Iron	100 ± 42 (mg/kg)
Zinc	62 ± 12 (mg/kg)
Manganese	6 ± 7 (mg/kg)
Copper	1 ± 1 (mg/kg)

**Table 5:** Examples of conventional extraction methods for seafood waste valorization.

Valorized Products	Source	Extraction Method	Main Outcome	Application	Ref.
Collagen	Tilapia, grey mullet (skin/scales)	Acid/alkaline extraction with pre-treatment and precipitation	40% collagen recovery	Wound healing and tissue repair	[42]
Fish oil	*Cyprinus carpio* (common carp) viscera	Thermal extraction and acid fermentation	Oil recovery of 57.14% (boiling) and 35.71% (acid fermentation)	Edible oil production	[43]
Chitin	White shrimp shells	Acid demineralization and enzymatic deproteinization	29–30% chitin recovery	Biopolymer production	[44]
Nano-hydroxyapatite/chitosan biocomposite (nHCB)	Fish bones (Catfish) and shrimp shells	Enzymatic hydrolysis using Alcalase	High pollutant adsorption performance	Wastewater treatment and pollutant removal	[45]
Gelatin, fish bone and scale flour	Fish scales and bones	Acid pretreatment and thermal extraction	High protein content and favorable sensory properties	Functional food ingredients	[46]
Calcium phosphate	Fish bone (tilapia carcass)	Acid-base precipitation and calcination	Biphasic nanostructured calcium phosphate material	Bone regeneration and orthopedic applications	[47]

**Table 6:** Applications of emerging technologies for the extraction of bioactive compounds from seafood by-products.

Valorized Products	Source	Extraction Method	Main Outcome	Application	Ref.
Bioactive lipids (EPA, DHA)	Carp caviar, viscera, fillet	Supercritical CO_2_ extraction	High recovery of omega-3-rich lipids	Functional foods	[48]
Fish oil rich in EPA and DHA	Mackerel muscle	Supercritical CO_2_ extraction	High-quality oxidatively stable oil	Nutraceuticals, functional foods, encapsulated delivery systems	[49]
PUFA-rich oil	Lobster liver	Supercritical CO_2_ extraction	PUFA-rich edible oil	Food and nutraceuticals	[50]
Astaxanthin	Crawfish tail shells	Supercritical CO_2_ with ethanol cosolvent	High antioxidant pigment recovery	Aquaculture, nutraceuticals	[51]
Oligochitosan	Shrimp shells	Subcritical water hydrolysis	Bioactive low-MW chitosan derivatives	Biomedical, pharmaceutical, functional food ingredients	[52]
MUFA/PUFA-rich oil	Fish head	Microwave + ultrasound-assisted enzymatic extraction	High-yield fish oil with improved stability	Nutraceuticals, functional foods	[53]
Collagen	Sea bass skin	Ultrasound-assisted extraction	Enhanced collagen recovery	Food, cosmetics, biomedical	[54]

**Table 7:** Comparative overview of extraction technologies used in seafood waste valorization.

Method	Main Target Compounds	Advantages	Limitations	Environmental Impact
Solvent extraction	Lipids	High extraction efficiency	Toxic solvents, solvent recovery required	High
Enzymatic hydrolysis	Proteins, peptides	Mild and selective processing conditions	High enzyme cost, long processing time	Moderate
Supercritical fluid extraction (SFE)	Lipids, omega-3 fatty acids	Solvent-free products, high-quality oils	High equipment and operating cost	Low
Pulsed electric field (PEF)	Intracellular bioactive compounds	Non-thermal process, reduced processing time	Limited industrial application	Low
Ultrasound-assisted extraction (UAE)	Proteins, lipids, collagen	Reduced solvent and energy consumption	Possible degradation of sensitive compounds	Low
Microwave-assisted extraction (MAE)	Lipids, proteins, bioactive compounds	Rapid extraction and shorter processing time	Risk of non-uniform heating	Moderate
Subcritical water extraction (SWE)	Proteins, peptides, lipids, chitin derivatives, bioactive compounds	Green solvent, simultaneous extraction and hydrolysis, suitable for wet biomass	High-pressure operation, possible thermal degradation at severe conditions	Low

**Table 8:** Quantitative summary of typical operating conditions, products, yields, selectivity, and severity effects for major product classes obtained from seafood by-products using subcritical water.

Product Class	Typical Operating Conditions	Main Products	Typical Yields	Product Selectivity (Favourable Conditions)	High Severity Effect	Ref.
Proteins, peptides, and amino acids	140–260 °C, 20–100 bar, 5–60 min	Protein hydrolysates, low-MW peptides, antioxidant peptides, free amino acids	Protein recovery typically 50–90%; near-complete solubilization reported; amino acid production strongly feedstock-dependent	160–200 °C: peptide-rich extracts and high antioxidant activity. 180–220 °C: increased free amino acid formation.	>200–220 °C amino acid degradation, Maillard reactions, browning, loss of bioactivity	[6,7,8,9,10,12,65,78,79,80,81]
Lipids/Fish oil	180–300 °C, 30–100 bar, 10–60 min	Fish oil, free fatty acids, EPA, DHA	Oil recovery typically 60–90%; feedstock dependent	200–240 °C: oil recovery with EPA/DHA preservation	>240 °C: PUFA oxidation and thermal degradation	[65,82,83,84,85]
Collagen/Gelatin	120–250 °C, 20–100 bar, 5–60 min	Gelatin, collagen peptides	Gelatin recovery typically 50–80%	140–180 °C: gelatin; 200–250 °C: collagen peptides	>250 °C excessive depolymerization and reduced functionality	[6,8,81,86,87]
Chitin/Chitosan derivatives	170–260 °C, 30–100 bar, 5–30 min	α-Chitin, chitin oligomers, glucosamine	Highly dependent on shell composition; improved purity via deproteinization	240–260 °C: high-purity chitin	>260 °C excessive severity: depolymerization, 5-HMF, levulinic acid, char	[11,71,72,73]
Mineral-rich products	160–240 °C, 30–100 bar, 20–120 min	Hydroxyapatite, calcium phosphates	High mineral recovery from bones and shells	180–220 °C mineral enrichment	Excess severity: reduced crystallinity, mineral dissolution	[8,11,24,32,33,47]

**Table 9:** Summary of some of the works on seafood waste valorization using SWE.

Feedstock	Process	Conditions	Products and Yield	Application	Ref.
SWE					
Tuna fish meal	SWE	140–180 °C, 50 bar, 300 min	Free AA 344 mg/g protein; peptides 275 mg/g protein	Food industry	[78]
Fish waste (mixed ocean fish)	SWE	140–220 °C, 30 bar, 5 min	Protein 1.705 g/L	Animal feed production	[7]
Fish meat (horse mackerel)	SWE	200–400 °C, 15–160 bar, 1–30 min	AA up to 0.024 kg/kg dry meat	-	[65]
Cod frames	SWE	90–250 °C, 100 bar, 30 min	Proteins, peptides and amino acids 57.7 g/100 g	Food and nutraceutical applications	[8]
Squid waste	SWE	170–400 °C, 8–300 bar, 1–50 min	AA 0.055 kg/kg; organic acids 0.055 kg/kg	-	[9]
Squid muscle	SWE	160–280 °C, 6–66 bar, 3 min	Free and structural AA 421 mg/100 g, 380 mg/100 g	Natural additives in food applications	[79]
Fish gelatin (cold-water fish)	SWE	160–240 °C, 20 bar, 157 min	Max AA at 220 °C	Raw materials of functional foods	[86]
Shrimp cephalothorax	SWE	230–280 °C, 20–50 bar, 30 min	Chitin 82.2 wt%	-	[11]
Abalone viscera	SWE	110–230 °C, 60 min	Protein and carbohydrates yield 46%	-	[12]
Mackerel bone and skin	SWE	200–250 °C, 30–70 bar, 3 min	Pepsin-solubilised collagen up to 8.1%	Functional ingredient in the food, cosmetic and pharmaceutical industries	[87]
Tuna skin and collagen	SWE	120–300 °C, 50–100 bar, 5 min	Bioactive peptides, Max antioxidant activity at 280 °C	Functional elements in food industries	[6]
Combined processes					
Yellow corvina (head/viscera)	SC-CO_2_ + SWE	27 g/min, 3 h + 160–235 °C, 15 min	Oil 7.6–15.5%; AA max 235 °C	Functional food development	[83]
Sardine waste	SC-CO_2_ + SWE	40 °C, 250 bar + 90–250 °C, 100 bar, 30 min	Oil 20.3 g/100 g; ω-3 up to 17.2%, highest antioxidant activity at 250 °C	Food, pharmaceutical, and cosmetic industries	[85]
Conger eel by-products	SC-CO_2_ + SWE	27 g/min, 55 °C, 300 bar, 2 h + 160–280 °C, 40 bar, 15 min	Protein 409 mg/g	Functional materials	[84]
Blue mussel	SC-CO_2_ + SWE	27 g/min, 55 °C, 300 bar, 2 h + 120–240 °C, 30 bar, 30 min	Free AA 11,718 mg/L (max)	-	[80]
Tilapia scales	SWE+ enzymatic treatment	85–135 °C, 15–120 min	Protein recovery 84.81%	Food ingredients	[81]
Fish waste (Northern anchovy, salmon, and cod) + shrimp waste (pink, tiger, and brown)	HTL +enzymatic + microwave	150–210 °C, 60–120 min	Bio-crude Liquor and Bio-oil	-	[93]
HTL and HTC processes					
Waste shrimp shell	HTC	180 °C, 12 h	Hydrochar	Adsorbent candidate for anionic dye removal	[89]
fish waste (heads, tails, viscera, fins, and scales from northern anchovy, salmon, and cod)	HTC	150–220 °C, 60–120 min	Biochar yield 35%	Energy generation	[90]
Small yellow croaker	HTL	200–300 °C, 95–185 min,	Biocrude oil yield 47%	Fuel and energy applications	[91]
Anglerfish waste	HTL	200–295 °C, 30–60 min	Biocrude oil yield 68.8%	Energy supplementation	[92]

## Data Availability

No new data were created or analyzed in this study. Data sharing is not applicable to this article.

## Abbreviations

The following abbreviations are used in this manuscript:

SWE	Subcritical water extraction
HTC	Hydrothermal carbonization
HTL	Hydrothermal liquefaction
FAO	Food and agricultural organization
COD	Chemical oxygen demand
BOD	Biological oxygen demand
SFA	Saturated fatty acid
MUFA	Monounsaturated fatty acid
PUFA	Polyunsaturated fatty acid
EPA	Eicosapentaenoic acid
DHA	Docosahexaenoic acid
GaG	Glycosaminoglycan
HA	Hyaluronic acid
SFE	Supercritical fluid extraction
PEF	Pulsed electric field
UAE	Ultrasound-assisted extraction
MAE	Microwave-assisted extraction
UV	Ultraviolet (light)
MW	Molecular weight
FFA	Free fatty acid
NAG	N-acetyl-D-glucosamine
5-HMF	5-hydroxymethylfurfural
PSC	Pepsin solubilized collagen
CHTC	Conventional hydrothermal carbonization
MHTC	Microwave-assisted hydrothermal carbonization
GC-MS	Gas chromatography–mass spectrometry
SC-CO_2_	Supercritical carbon dioxide
ACE	Angiotensin I-converting enzyme
AA	Amino acids
LCA	Life cycle assessment
TEA	Techno-economic analysis
